# Structure of methyltransferase RedM that forms the dimethylpyrrolinium of the bisindole reductasporine

**DOI:** 10.1016/j.jbc.2023.105520

**Published:** 2023-11-30

**Authors:** Phillip Daniel-Ivad, Katherine S. Ryan

**Affiliations:** Department of Chemistry, The University of British Columbia, Vancouver, Canada

**Keywords:** methyltransferase, indolocarbazole, biosynthesis, crystal structure, enzyme catalysis, site-directed mutagenesis

## Abstract

Bisindoles are biologically active natural products that arise from the oxidative dimerization of two molecules of l-tryptophan. In bacterial bisindole pathways, a core set of transformations is followed by the action of diverse tailoring enzymes that catalyze reactions that lead to diverse bisindole products. Among bisindoles, reductasporine is distinct due to its dimethylpyrrolinium structure. Its previously reported biosynthetic gene cluster encodes two unique tailoring enzymes, the imine reductase RedE and the dimethyltransferase RedM, which were shown to produce reductasporine from a common bisindole intermediate in recombinant *E. coli*. To gain more insight into the unique tailoring enzymes in reductasporine assembly, we reconstituted the biosynthetic pathway to reductasporine *in vitro* and then solved the 1.7 Å resolution structure of RedM. Our work reveals RedM adopts a variety of conformational changes with distinct open and closed conformations, and site-directed mutagenesis alongside sequence analysis identifies important active site residues. Finally, our work sets the stage for understanding how RedM evolved to react with a pyrrolinium scaffold and may enable the development of new dimethyltransferase catalysts.

Bacterial bisindoles are a group of alkaloid natural products that arise from the oxidative dimerization of l-tryptophan (1, 2). The first bisindole isolated was the purple pigment violacein in 1882 (3), and the discovery of the potent kinase inhibitor staurosporine in 1977 (4) led to huge interest in the large class of indolocarbazole natural products as drug leads starting in the late 1980s (5) (Fig. 1*A*). Biosynthetic studies beginning in the 2000s revealed that bacterial indolocarbazoles arise from the oxidative dimerization of l-tryptophan through three key steps: (1) oxidation to indole pyruvate imine; (2) oxidative dimerization to give chromopyrrolic acid, and (3) aryl-aryl coupling to give the indolocarbazole structure (Fig. 1*C*) (6, 7, 8). A combination of enzyme-mediated and non-enzymatic oxidation then gives the core aglycone structure observed in diverse bisindoles (8, 9). To target new bacterial bisindole gene clusters and their encoded metabolites, Brady and co-workers developed degenerate primers specific to the chromopyrrolic acid synthase genes to enable the targeted isolation of bisindole biosynthetic gene clusters from environmental DNA (10). Using these primers, they isolated novel biosynthetic gene clusters, and through various reconstitution techniques, reported a number of new bisindole structures, including erdasporine and reductasporine (Fig. 1*A*) (11).

**Figure 1:**
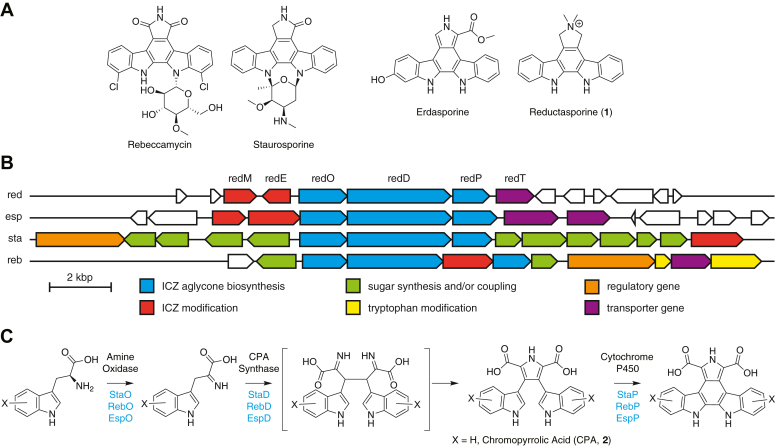
**Biosynthesis of reductasporine.***A*, natural product indolocarbazoles. *B*, gene clusters for reductasporine (*red*), erdasporine (*esp*), staurosporine (*sta*), and rebeccamycin (*reb*). Indolocarbazole is abbreviated ICZ. *C*, biosynthetic assembly of indolocarbazoles.

Reductasporine has an unusual dimethylpyrrolinium structure whose synthesis is linked to the six-gene cluster, *redODPEMT* (Fig. 1*B*) (11). In a previous study, no new metabolites were observed after transformation of heterologous hosts with a cosmid containing the full gene cluster, and the result did not change by introducing an inducible T7 promotor in front of each gene. However, replacing the core indolocarbazole genes, *redODP*, with *espODP* from the erdasporine gene cluster, known to express well in *E. coli* (12), and co-expressing with *redE* and *redM* under the T7 promoter created recombinant strains that produce reductasporine. Based on this result, Chang and coworkers put forward a biosynthetic proposal whereby RedE intercepts an unstable intermediate product, 12,13-dihydro-6*H*-indolo[2,3-*a*]pyrrolo[3,4-*c*]carbazole (**4**), from the reaction of the cytochrome P450 EspP with CPA. RedE reduces compound **4** to didemethylreductasporine (**3**), and then RedM catalyzes two methylations to give reductasporine (Fig. 2*A*). We became interested in both RedE and RedM, as they are unusual biosynthetic enzymes among the arsenal of indolocarbazole enzymes. In particular, RedM is a rare *N,N-*dimethyltransferase. Here we report the *in vitro* activity of RedE and RedM and solve a 1.7 Å resolution structure of RedM in complex with each SAM and SAH. Our work reveals a large conformational change in RedM that may be relevant to catalysis, along with identifying key active site residues.

**Figure 2:**
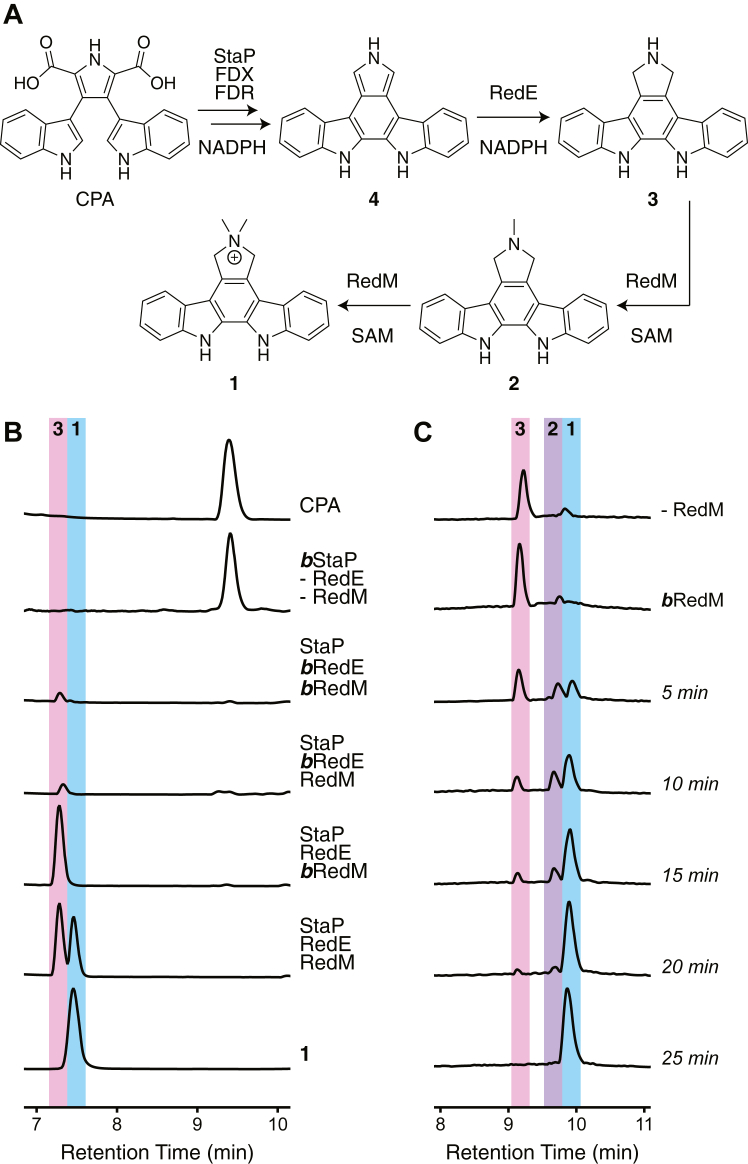
***In vitro* reconstitution of reductasporine biosynthesis.***A*, proposed pathway for the formation of reductasporine from chromopyrrolic acid. *B*, HPLC-MS traces (base peak count) of 300 μM CPA, 10 mM NADPH, 3 mM SAM, 5 μM StaP 10 μM ferredoxin, 1 μM ferredoxin reductase and 50 mM HEPES pH 8 in the presence of active and/or boiled (denoted as ***b***) RedE and RedM (20 μM each). *C*, methylation of synthetic **3** to reductasporine by RedM (2 μM RedM 2 mM SAM and 500 μM **3** over 25 min).

## Results

### *In vitro* reconstitution of reductasporine biosynthesis

To provide evidence for the accumulation of proposed reductasporine biosynthetic intermediates and the functions of enzymes RedE and RedM, we sought to reconstitute the pathway with purified enzymes (Fig. S1). We isolated chromopyrrolic acid (CPA) from the cleared *E. coli* lysates containing RebO and RebD incubated with tryptophan. We purified RedE, RedM, cytochrome P450 StaP, *Synechococcus elongatus* ferredoxin (UniProt ID P0A3D2), and ferredoxin reductase (UniProt ID Q31PL1), each recombinantly expressed in *E. coli* (13). In control reactions, we observe that StaP together with ferredoxin, ferredoxin reductase, and NADPH consumes chromopyrrolic acid, as expected. The inclusion of RedE results in the accumulation of a new product ([M + H]^+^ = 298), with a mass corresponding to **3**. The inclusion of both RedE and RedM yields reductasporine as verified *via* NMR (Figs. 2*B* and S2). To further interrogate the reaction, we directly incubated synthetic **3** with RedM with SAM and observed *via* LC-MS analysis the accumulation and disappearance of a peak consistent with the monomethylated intermediate (**2**) over 25 min (Fig. 2*C*), providing initial evidence that the dimethylation occurs over discrete steps.

### Crystal structure of RedM

To provide insight into the N,N-dimethylation catalyzed by RedM, we solved the structure of selenomethionine-labeled RedM, and used this model to solve crystal structures of unliganded enzyme, as well as a co-crystal structure with SAH and a co-crystal structure with SAM, all to 1.7 to 1.85 Å resolution (Table 1). Attempts to soak in or co-crystallize arcyriaflavin A, reductasporine or indolocarbazole precursor chromopyrrolic acid did not yield ligand-bound structures.

**Table 1:** Data collection and refinement statistics^a^

	Unliganded RedM	RedM-SAH	RedM-SAM
Wavelength (nm)	0.97946	0.97946	0.97946
Resolution range (Å)	72.5–1.73 (1.76–1.73)	38.1–1.85 (1.89–1.85)	89.2–1.82 (1.85–1.82)
Space group	P 2_1_ 2_1_ 2_1_	P 2_1_ 2_1_ 2_1_	P 2_1_
Unit cell	61.95 71.61 145.00	61.83 71.90 144.84	113.86 58.78 113.92 90,103.11 90
Total reflections	778,150 (21,218)	729,957 (44,012)	823,931 (41,491)
Unique reflections	67,710 (3483)	55,851 (3356)	125,933 (6228)
Multiplicity	11.5 (6.1)	13.1 (13.1)	6.5 (6.7)
Completeness (%)	99.4 (95.0)	99.9 (98.4)	95.9 (96.7)
Mean I/sigma(I)	23.6 (5.8)	15.4 (1.2)	9.5 (1.6)
Wilson B-factor	17.89	38.1	21.01
R_merge_	0.065 (0.241)	0.068 (1.703)	0.146 (1.215)
R_meas_	0.067 (0.263)	0.070 (1.772)	0.159 (1.316)
R_pim_	0.019 (0.101)	0.019 (0.485)	0.061 (0.501)
CC_1/2_	0.999 (0.960)	0.999 (0.730)	0.997 (0.367)
Reflections used in refinement	67,168 (6471)	55,630 (5406)	125,905 (12,596)
Reflections used for R_free_	3390 (337)	1993 (193)	6531 (622)
R_work_	0.1669 (0.2045)	0.1969 (0.3635)	0.1753 (0.2946)
R_free_	0.1992 (0.2339)	0.2285 (0.3896)	0.2175 (0.3301)
Total non-hydrogen atoms	5726	5084	11,226
macromolecules	4835	4728	9814
ligands	36	11	179
solvent	855	345	1233
Protein residues	631	631	1297
RMS (bonds)	0.004	0.005	0.005
RMS (angles)	0.68	0.75	0.77
Ramachandran favored (%)	98.39	98.23	97.58
Ramachandran allowed (%)	1.61	1.77	2.42
Ramachandran outliers (%)	0.00	0.00	0.00
Rotamer outliers (%)	0.21	0.68	0.73
Clashscore	1.86	1.50	2.68
Average B-factor	21.98	45.45	30.81
macromolecules	19.93	45.17	29.98
ligands	31.15	57.83	37.63
solvent	33.24	48.87	36.47
Number of TLS groups	14	4	17

a Statistics for the highest-resolution shell are shown in parentheses.

**Figure 3:**
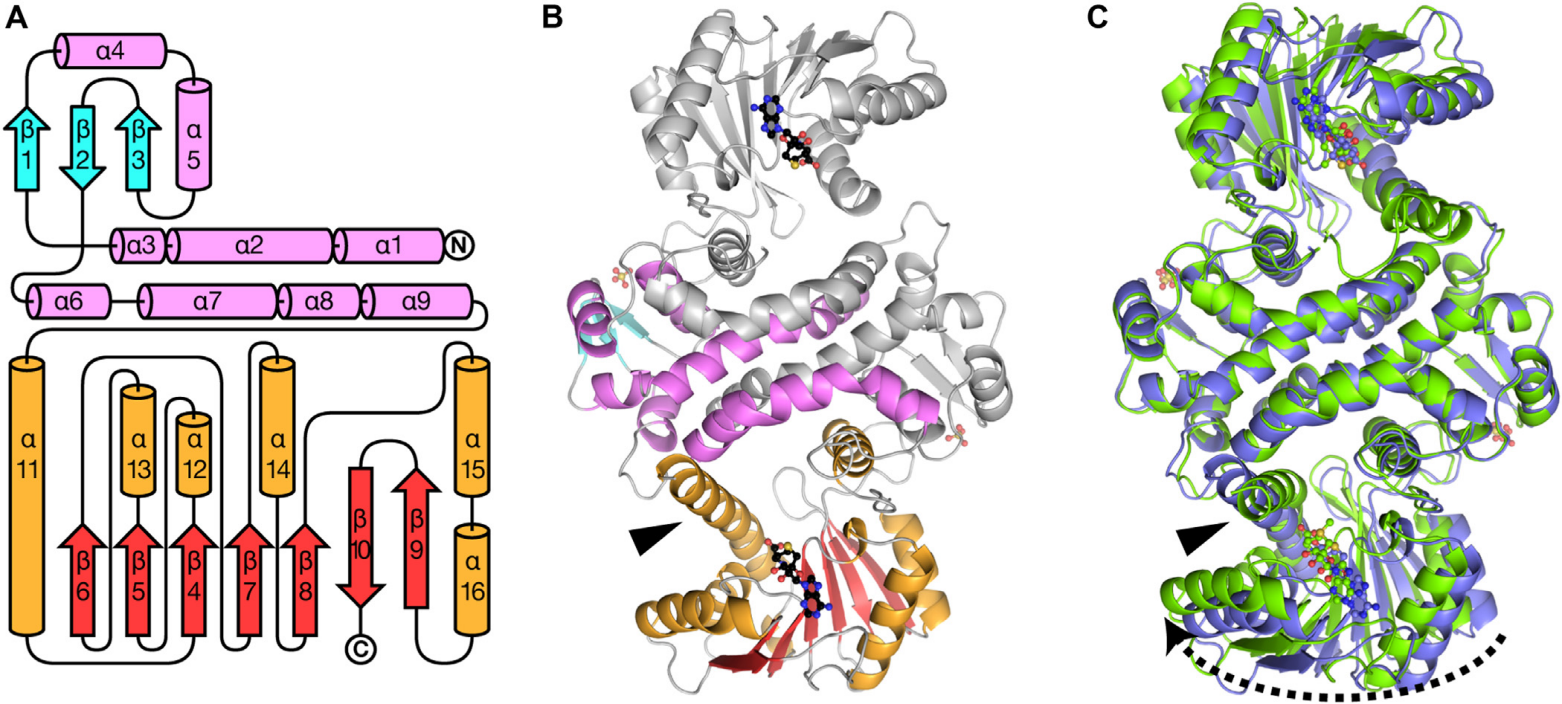
**Structure of RedM.***A*, topology diagram with labelled secondary structures. N-terminal dimerization domain highlighted in *pink* and *cyan*, and C-terminal Rossmann-like SAM-binding domain coloured *orange* and *red*. Helix *α*10 is omitted from the diagram as it is not found on all protomers and borders the gap between the N-terminal domain and interdomain helix. *B*, overall fold of RedM with SAH bound (*black*). The domain bridging *α*11 helix is indicated with a triangle. *C*, redM-SAH and -SAM binary complexes superposed by their dimerization domains. One protomer of each dimer in the RedM-SAM crystal structure asymmetric unit shows a distinct deviation from the more open conformation seen in the RedM-SAH complex. The more constricted, closed conformation arises from the Rossmann-like domain’s rotation towards the dimerization domain (*dashed arrow*) and a substantial deflection of the interdomain *α*11 helix (*triangle*).

RedM is a homodimeric protein with monomers consisting of a largely *α*-helical N-terminal domain (*α*1-10, *β*1-3) and a Rossmann-like fold C-terminal domain (*α*12–16, *β*4-10) bridged by a long interdomain *α*-helix (*α*11) (Fig. 3*A*). Size-exclusion chromatography supports that RedM exists as a homodimer in solution (Fig. S3). The N-terminal domains of the homodimer form the majority of the dimer interface with the only contact with the C-terminal domain occurring between *α*5, *α*8, *α*9 (residues 62–73, 112–125), and *α*15′ (residues 292–304). The extensive dimer interface is approximately 3150 Å^2^, accounting for nearly one-fifth of the total monomer surface area, as determined by PISA (14). These buried residues in the interface are primarily hydrophobic, and the few polar residues are small serine or threonine forming hydrogen bonds to nearby peptide bonds. The two domains of each monomer are separated by a gap of ∼12 amino acids between residues ∼132 and ∼144 ahead of the long interdomain *α*-helix (*α*11), which lacks clear electron density in each structure. In RedM-SAM co-crystal structures *α*11 adopts a pronounced kink at either M157 or M162 in several protomers. The C-terminal domain possesses a Rossmann-like fold typical of Class I SAM-dependent methyltransferases, with a *β*-sheet of five parallel strands terminated with two antiparallel strands each interspersed with *α*-helices (15, 16, 17). SAM binds in the structurally conserved cleft between the sharp turn after *β*4 and the start of the *β*5 strand.

Secondary structure matching searches using PDBeFOLD (18) ranks RedM as most related to other SAM-dependent enzymes acting on small molecules, all of which are part of the caffeic acid O-methyltransferase-type family (COMT-type, IPR016461). The full-length monomer of RedM bears the greatest structural similarity to the human N-acetylserotonin O-methyltransferase (ASMT, PDB: 4A6E, 29% identity, rmsd_C*α*_ = 1.6 Å, 291 C_*α*_). Other highly ranked structural homologs include PigF (7CLF), an O-methyltransferase involved in prodigiosin biosynthesis (19); PdxI (7BQK), a pericyclase related to the LepI pericyclase in leporin biosynthesis (20, 21); Ca9OMT (4E70); an O-methyltransferase modifying the lignin precursor coniferyl alcohol (22); BchU (1X19), a C-methyltransferase involved in bacteriochlorophyll C biosynthesis (23); and PhzM (2IP2), an N-methyltransferase part of the pyocyanin biosynthetic pathway (24). Each retains the glycine-rich sequence along with the neighboring acidic residue indicative of the SAM binding cleft (15, 16, 17).

The homodimers of both unliganded-RedM and RedM-SAH structures superpose well on each other (rmsd_C*α*_ = 0.175 Å, 553 C_*α*_) and comparatively poorly upon the RedM-SAM homodimer (rmsd_C*α*_ = 1.244 Å, 593 C_*α*_) due to a substantial displacement of the C-terminal Rossmann-like SAM-binding domain (Figs. 3*C* and S4*A*). The movement is an approximate 12° rotation toward *α*11 about an axis aligned with *α*15, where the loop regions around *α*15 act as flexible hinges. Residues furthest from *α*15 are displaced up to 7 Å. Helix *α*11 accommodates this conformational change by developing a kink at M157 or S161 with 31 to 49° changes in the Phi and Psi angles at these residues. The overall length of the monomer is reduced by 2.5 Å and the central binding cavity volume is reduced by up to 60% (estimated *via* POCASA (25)), and likely represents a closed conformation of RedM. This conformation is seen in two protomer chains of the RedM-SAM structure: chains A and B. They adopt slightly different closed conformations with an average C_*α*_ displacement of 2.5 Å, rotating toward the dimerization domain about an axis roughly parallel with helix *α*8. Chains C and D have more open conformations similar to the unliganded and SAH-bound RedM structures. Furthermore, chain C is further rotated away from the dimerization domain with an average C_*α*_ displacement of 3 Å compared to RedM-SAH chain A (Fig. S4*B*). Lastly, Chain B of the RedM-SAH complex and unliganded-RedM chain A have slightly rotated Rossman-like domains compared to their dimer partners with an average C_*α*_ displacement of 1.8 Å between each pair. The centers of mass of the Rossman-like domains capture the relative motions between chains (Fig. S5). The difference in C_*α*_ displacements between the open-to-open conformations (RedM-SAH chain A to RedM-SAM chain C) and closed-to-closed conformations (RedM-SAM chain A to chain B) is remarkably similar. The magnitude of the relative displacement between C_*α*_’s averages 1.1 Å (RMSD = 1.2 Å). Similarly, the differences between open-to-closed conformations (RedM-SAH chain A to RedM-SAM chain A or RedM-SAM chain C to RedM-SAM chain B) with the magnitude of the relative displacement between C_*α*_’s averages 0.9 Å (RMSD = 1.0 Å).

**Figure 4:**
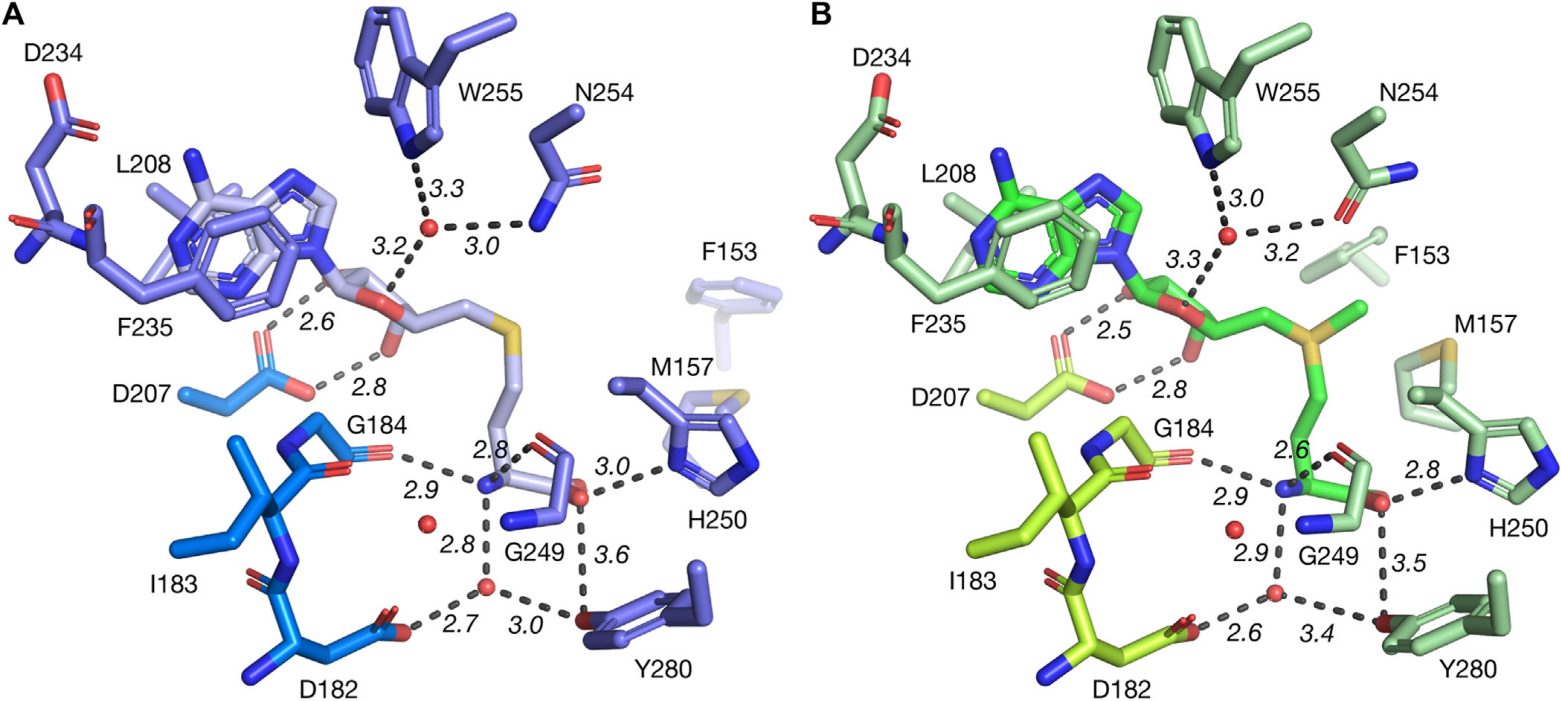
**RedM active site residue contacts.***A*, SAH in the open conformation and (*B*) SAM in the closed conformation. The conserved SAM-binding motifs I-II (D182-G184, D207) are highlighted and form key hydrogen bonds to the *α*-amine and ribosyl moiety. The closed conformation brings residues F153 and M157 to within 4 Å of the active methylsulphur moiety.

The cofactor binding pocket is outlined by the C-terminal ends of *β*4-8 and clear electron density shows the cofactor is held in an extended conformation similar to related small molecule methyltransferases (15, 16, 17). These Class I methyltransferases bear the conserved E/DxGxGxG SAM-binding motif and is found as 182-DIGCAEG-188 in RedM. The main chains of C185, A186, and G188 form the foundation of the cavity between the ribosyl and methionine moieties, while D182 hydrogen bonds to two conserved water positions creating the basis for a network of hydrogen bonds bridging the *α*-amine to I183, Y280, and G249 *via* its amide nitrogen (Fig. 4). The carbonyl groups of G184 and G249 receive hydrogen bonds from the *α*-amine, while H250 and Y280 donate hydrogen bonds to the cofactor carboxylate group. The ribosyl hydroxyl groups interact closely with D207, representing the second conserved binding motif in SAM-dependent methyltransferases (17). N254 and W255 hydrogen bond a third conserved water position which bridges to the furan oxygen. The adenine ring is clamped between F235 and L208 and capped with a hydrogen bond to D234. Upon closure of the active site, residues F153 and M157 from the interdomain helix *α*11 are brought to within 4 Å of the SAM methylsulfur, displacing the solvent (Fig. 4*B*). The SAM-binding contacts do not change between both chains displaying closed conformations in the RedM-SAM structure, with the exception that F153 in chain B does not have fully resolved electron density of the phenyl side chain.

### Modeling the RedM-substrate complex

Despite efforts to co-crystallize RedM with reductasporine or substrate analogs, no clear electron density for additional ligands was resolved. We, therefore, focused on modeling the RedM-substrate complexes *via* docking experiments, noting that the rigidity of the indolocarbazole skeleton might limit the substrate to a single conformation.

**Figure 5:**
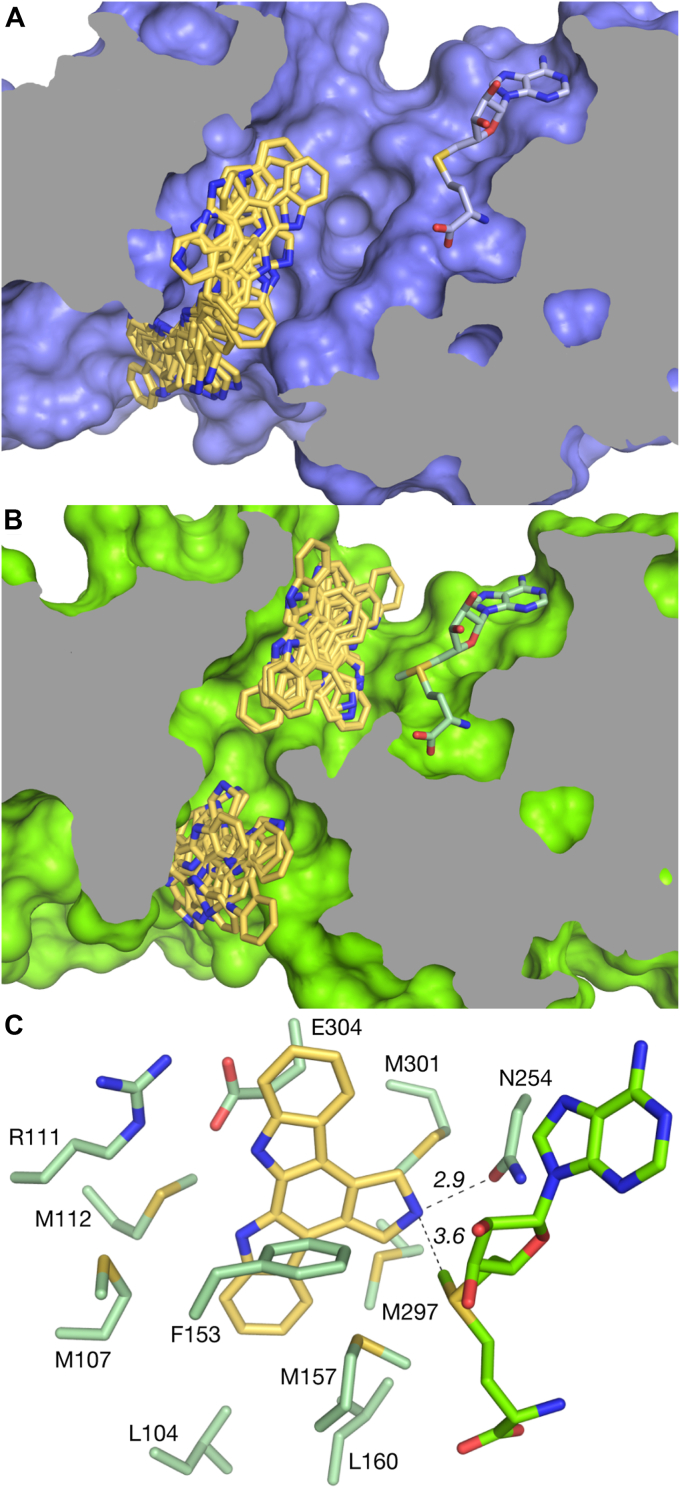
**Results from docking didemthylreductasporine (3) into the open and closed conformations of RedM.** Docking search space spans the entire internal cavity. *A*, the open conformation shows no favourable binding in the vicinity of SAH and poses aggregate among the hydrophobic residues of the N-terminal dimerization domain. *B*, docked poses in the closed conformation show that constriction of the binding cavity forms a pocket for indolocarbazole binding. Several poses have favorable geometry for methyl transfer. *C*, active site residues surrounding a docked **3** pose with pyrroline nitrogen positioned in line with and 3.6 Å away from the SAM methylsulfur group. 3 is docked to the closed conformation of RedM and the N254 sidechain was allowed to rotate freely during docking. Five methionine residues and F153 pack closely with the indolocarbazole aromatic ring system. Distances are shown in units of Angstrom.

Docking search with **3** on the open conformation places moderately favorable poses at the junction of helices *α*7, *α*11, *α*15, and *α*′1 and *α*ʹ2 of the dimer partner, an average of 20 Å away from the bound SAH cofactor (Fig. 5*A*). This junction is lined with mainly hydrophobic residues (L18, M25, L104, M112, P293, L296, M297, P336), and several small polar residues (D15 Q19, T22, S108, T300). D15 may be engaged in a salt bridge to R163 joining the N-terminus to the interdomain helix (*α*11); however, this interaction only has unambiguous electron density in one monomer of the RedM-SAH complex. In docking the substrate to the closed conformation several poses remain in this distal area, but higher-scoring poses occur near SAM (Fig. 5*B*). These poses have strained geometry between the donor methylsulfur and acceptor nitrogen for methyl transfer. Allowing free rotation of the N254 side chain in the docking procedure yields binding conformations with near-ideal geometry (Fig. 5*C*). Docking **2** under identical settings results in poses with close proximity to enable methyl transfer, but non-ideal geometry. Introducing free rotation in neighboring residues S253, M297 and M301 yielded more poses in the vicinity of SAM with similar geometry.

Using these docking results as a guide we describe a putative indolocarbazole binding site (Fig. 5*C*). The cavity is located at the junction of helices *α*7, *α*8, *α*11, and *α*15 and the loop between *β*7 and *α*14, forming a roughly 7 Å diameter hole through the center of the protomer in the open conformation. The area is lined with hydrophobic residues and is mostly methionine residues (L104, M107, M112, F153, M157, L160, M297, and M301). Methionine sulfur-*π* interactions are noted as stabilizing contacts with aromatic groups (26, 27). The closure of this cavity brings residues L160 and M297 within 4 Å of each other and forms the hydrophobic floor to the active binding pocket. R111 and E304 form a salt bridge overtop the cavity and join to *α*15 to the N-terminal helical bundle. N254 is in close proximity to the pyrroline nitrogen of the indolocarbazole substrate and may hydrogen bond in some conformations. The complete active site may include the residues with unresolved electron density in the range from 133 to 149, which form two *α*-helices capping the SAM-binding cavity in similar methyltransferase structures (24, 28, 29, 30).

### Mutagenesis of the RedM active site

**Figure 6:**
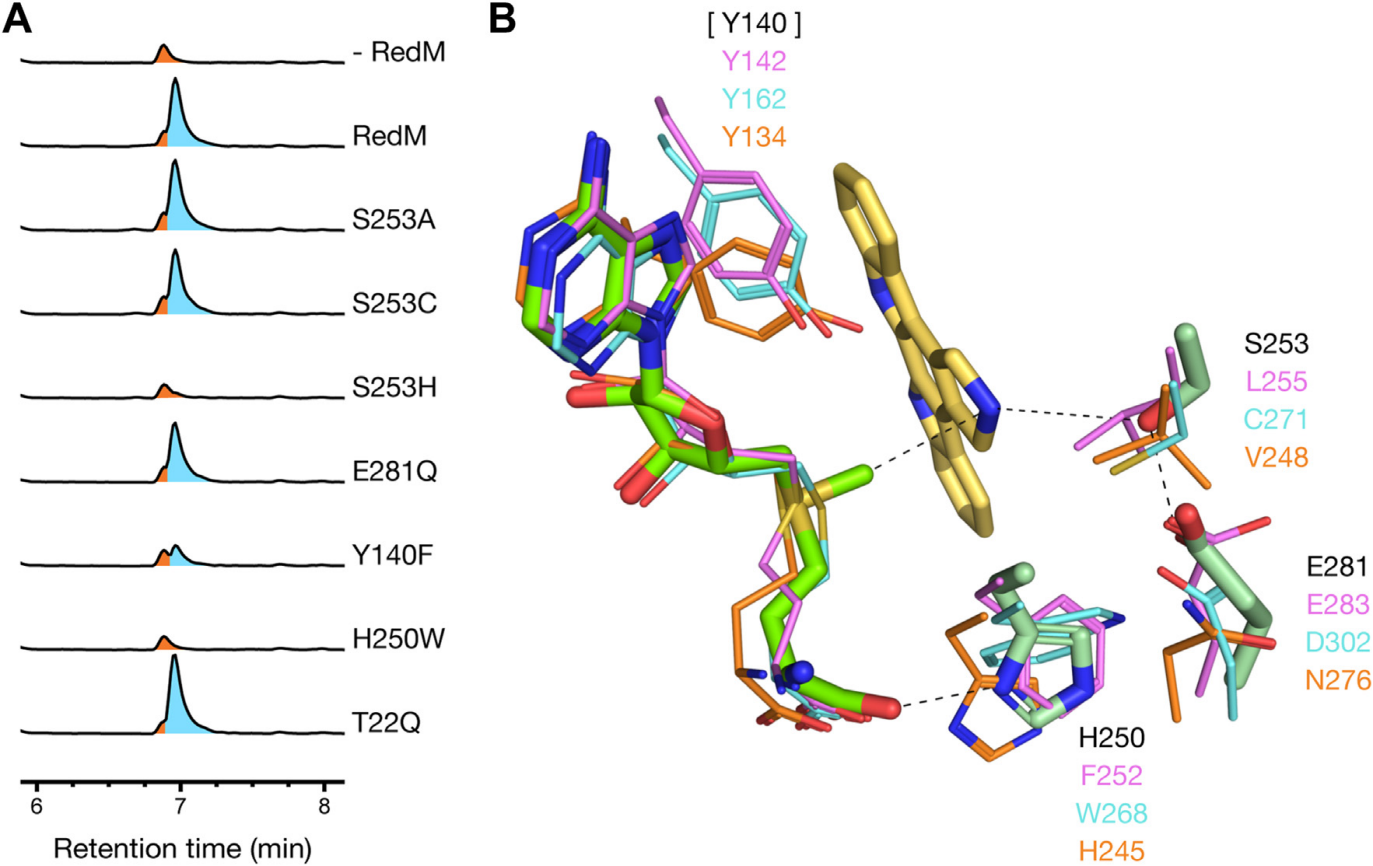
**Analysis of RedM variants.***A*, base peak LC-MS traces of CPA+StaP+RedE+RedM variant reaction mixes incubated at room temperature. Complete conversion to reductasporine (m/z = 326, *blue*) is seen after 1 h alongside a background peak (m/z = 310, *orange*). Complete loss in product accumulation is seen in RedM-S253H and -H250W and partial loss in RedM-Y140F. *B*, active site docking of didemethylreductasporine (**3**) with residues targeted for mutation rendered as light *green* sticks. Equivalent residues and cofactors in DnrK (*pink*, 1TW2), CA9OMT (*cyan*, 4EVI) and SibL (*orange*, 4U1Q) are overlayed for comparison. Y140 is not resolved in the structural models of RedM; however, its position may be inferred from these related methyltransferases. Structures are aligned by the Rossmann-like domain (DnrK, RMSD = 0.99 Å, 133 C_*α*_; CA9OMT, 1.32 Å, 155 C_*α*_; SibL, 1.04 Å, 154 C_*α*_) and Y142, Y162 and Y134 from each are an average of 4.3 Å from the cofactor sulfur atom.

In structural homologs, the residue at position S253 in RedM is typically a histidine and has been shown to be involved in deprotonation, either deprotonating the substrate before methylation or deprotonating the resulting product (Fig. S6) (28, 31, 32, 33). Coniferyl alcohol 9-O-methyltransferase instead has a cysteine at this position, and this cysteine is implicated by mutagenesis as catalytically important, possibly taking part in acid-base chemistry (22). These residues catalyzing proton transfer are also associated with one or two neighboring aspartate or glutamate residues thought to position the active residue for proton abstraction or may participate as a catalytic partner (28, 31, 34, 35, 36, 37, 38). In RedM, these residues are E281 and E310, with E281 positioned ideally for H-bonding to S253, and with E310 more than 4 Å away from S253. We generated the S253A and S253C variants, neither of which altered product formation; however, S253H lost activity (Fig. 6*A*). Variant E281Q did not affect product formation.

Several other structurally characterized methyltransferases do not conserve the catalytic histidine, including carminomycin 4-O-MT (DnrK) (39), 3-hydroxykynurenine C-MT (SibL) (40), dimethylsulfoniopropionate S-MT (DsyB) (41), 8-demethyl-8-amino-d-riboflavin N-MT (RosA) (29) and phenazine N-MT (PhzM) (24). Mutational studies in SibL, DnrK, and DsyB indicate a tyrosine in the *α*-helix capping the active site is essential for activity in SibL and significant for activity in DnrK and DsyB (39, 40, 41). Aligning these enzymes with PhzM, RosA, and RedM shows that each conserves this tyrosine, and variant RedM Y140F results in a substantial loss in product formation (Fig. 6*A*).

Next, we sought to perturb the N,N-dimethylating activity of RedM. Docking indicates the pocket opposite the SAM cofactor may accommodate a methyl group during the second methyl transfer. It is lined by residues H250, S253, N254, E281, T282 M297, M301, and E310, and most of these residues are associated with substrate recognition or with potentially catalytic residues. We generated H250W, despite the association of the histidine with SAM-binding, as many plant O-methyltransferases conserve a tryptophan at this position, and the extra bulk may occlude a methyl from the adjacent pocket. The H250W variant had a complete loss of activity. Docking results suggest the area around *α*1 and *α*2 may provide a pre-catalysis staging area for indolocarbazoles before moving through the central tunnel to the active site (Fig. 5, *A* and *B*). T22, in the bend between *α*1 and *α*2, points directly into this area and mutation to a larger residue may interfere with substrate access. However, the T22Q variant has no impact on product formation (Fig. 6*A*).

### Sequence similarity network of COMT-type methyltransferases

**Figure 7:**
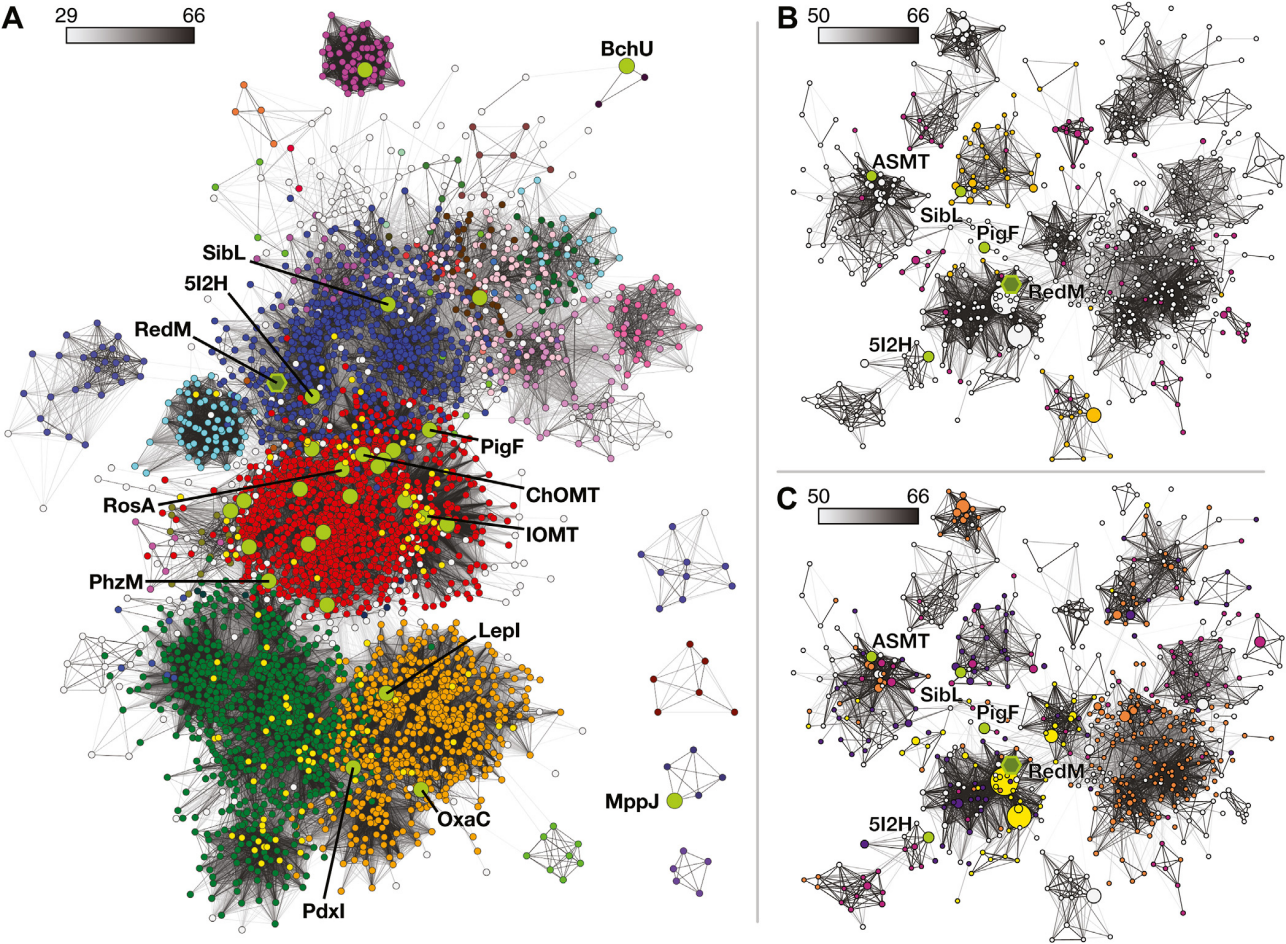
**Sequence similarity network of the COMT-like enzyme family (IPR016461) generated using the Uniref50 database** (95)**.***A*, visualization of the network with minimum edge alignment score of 29 and coloured according to the subclusters retained at an alignment score threshold of 50, inclusive. Most crystal structures (*bright green* nodes) are found in the largest subcluster alongside chalcone OMT and isoflavone-7-OMT, the founding members of the family (*red* nodes). The second largest subcluster (*green* nodes) contains fungal pericyclases such as PdxI (PDB: 7BQJ), transcriptional coactivators, and other methyltransferases. The third largest subcluster (*orange* nodes) is made up of mostly secondary metabolism methyltransferses such as OxaC (5W7P) and one example of a pericyclase in LepI (6IX5). RedM is found alongside human acetylserotonin MT (ASMT, 4A6E), SibL (4U1Q), demethylspheroidene O-methyltransferase (5I2H), and PigF (7CLF) (*blue* nodes). *B* and *C*, the cluster containing RedM isolated at an alignment score threshold of 50. *B*, *purple* and *orange* nodes have no catalytic histidine and *orange* nodes have a tyrosine residue corresponding to Y140 in RedM. This is a common catalytic motif in sequences related to SibL and is widely present in a nearby uncharacterized subcluster. This motif is only sporadically present in sequences related to RedM. *C*, nodes colored according to putative substrate recognizing positions 153-FxxxM-157/297-MxxxM-301 (RedM numbering) from least stringent (*purple*) to most stringent (*yellow*). Selections: [FL]xxx[ML]/xxxx[FML], *purple*; [FL]xxx[ML]/[FYML]xxx[FML], *maroon*; FxxxM/[FYML]xxxM, *orange*; FxxxM/MxxxM, *yellow*.

In order to visualize the distribution of active site motifs we created a sequence similarity network (SSN) of the COMT-type enzyme family (IPR016461) to which RedM belongs. The initial SSN at a permissive alignment score threshold (AST ≥29) divides the family into one main cluster and four small clusters (Fig. 7*A*). One includes MppJ, which binds iron in its active site in order to catalyze the unusual benzylic C-methylation of phenylpyruvate (42). This iron-binding site is unique in the COMT-type enzyme family. At a more restrictive alignment score threshold (≥50) the central cluster divides into four large clusters with more than 70 nodes. Most of the structurally characterized enzymes (19 of 41) are in the central, highly connected cluster and includes the first structurally characterized plant small molecule methyltransferases chalcone O-methyltransferase (ChOMT) and isoflavone-7-O-methyltransferase (IOMT) (28), and many methyltransferases participating in secondary metabolism (phenazines, bergaptol, scoulerine, flavonoids, and many others). This encompasses at least five methyltransferases that are capable of furnishing two methyltransfers: RosA (29), xanthohumol 4-O-methyltransferase (43), *trans*-resveratrol di-O-methyltransferase (44), myricetin O-methyltransferase (CrOMT2) (45), and 3-aminomethylindole N-methyltransferase (ANMT) (46). *β*-alanine N-methyltransferase (BANMT) is capable of methylating *β*-alanine up to three times (47). RedM occupies a more sparsely characterized cluster where the only structurally characterized enzymes are the C-methyltransferase SibL (catalyzing the C4 methylation of 3-hydroxykynurenine), O-methyltransferase PigF (part of prodigiosin biosynthesis), and human acetylserotonin O-methyltransferase (19, 40, 48). Other characterized proteins include the O-methyltransferase AviB2 (catalyzing methyl transfer to 3-hydroxy-5-methyl-1-naphthoate) and the O-methyltransferase TcmO (catalyzing methyl transfer to the 8-hydroxy in tetracenomycin biosynthesis) (49, 50). The remaining two large clusters of the network contain the majority of the eukaryotic COMT-type proteins primarily from fungal organisms. The first has 18 characterized natural product methyltransferases, which include enzymes involved in the biosynthesis of fumagillin, oxepinamides, phomasetin, and kotanin as well as the structurally characterized OxaC from oxaline biosynthesis (51) and the pericyclase-dehydratase LepI from leporin biosynthesis (52). The second cluster has the structurally characterized alder-ene pericyclases PdxI, EpiI and AdxI (from the putative pyridoxatin and fusaricide biosynthetic gene clusters) (21) along with 31 characterized natural product methyltransferases and a surprising 14 characterized transcriptional coactivators similar to the aflatoxin pathway regulator AflS (formerly AflJ) (53, 54). Overall, the characterized methyltransferases in this family are largely O-methyltransferases (78%) with N-methyltransferases the next most common (9%), and the remainder characterized as transcriptional coactivators, pericyclases, C-methyltransferases, and a single S-methyltransferase.

Examining the RedM subcluster at an AST of 50 or above shows some discrete separation in chemical activity (Fig. 7, *B* and *C*). For example, the C-methyltransferase SibL (4U1Q) and human acetylserotonin O-methyltransferase (4A6E) partition out into distinct clusters. The O-methyltransferase in prodigiosin biosynthesis, PigF, is not well connected and bridges several clusters near ASMT. Multiple sequence alignment and searches for the breadth of active site motifs show that it is common for methionine residues to be present in the active site, specifically at positions 157, 297, and 301 (RedM numbering). This includes the common FxxxM motif found in the interdomain helix and the MxxxM motif found in *α*15 in RedM (Fig. 7*C*). Lacking the histidine often considered catalytic (position 253) is uncommon, and when not present it is often supplemented with an alternative active site base, such as a tyrosine in the capping helix of the active site (Y140 in RedM). This tyrosine is nearly ubiquitous in the SibL subcluster, a different entirely uncharacterized subcluster nearby, and several proteins related to RedM (Fig. 7*B*).

## Discussion

*In vitro* reconstitution of the last steps in reductasporine biosynthesis revealed that RedE and RedM are independently functioning enzymes. RedE captures and reduces an unstable indolocarbazole intermediate, forming didemethylreductasporine (**3**), which is then a substrate for RedM. A time-course analysis with synthetic **3** reveals that RedM catalyzes the stepwise methylation of the RedE product, giving reductasporine. To provide insight into the structural basis for catalysis, we solved the structures of unliganded, SAH-, and SAM-bound RedM. Structural comparisons show RedM to be a typical member of natural product methyltransferases, classified in the InterPro database under the COMT-type methyltransferase family (IPR016461), possessing the characteristic N-terminal dimerization and C-terminal Rossmann-like domains (15, 16).

Between the RedM-SAH and RedM-SAM binary complexes, there are distinct open and closed conformations, respectively. The two chains in the asymmetric unit of the RedM-SAM complex adopting closed conformations have higher B-factors along the periphery of the Rossmann-like domain compared to their dimer partners and corresponding weaker crystal symmetry contacts (Fig. S7). The closed conformation may arise from weak crystal contacts allowing the enzyme to explore more conformational space and is biased towards a more closed conformation by interactions with SAM, or it may simply be trapped from solution in the growing crystal lattice. Nonetheless, each open and closed conformation is trapped in slightly different variations suggesting RedM has an inherent propensity for flexibility between its largely rigid N- and C-terminal domains. (Figs. S4 and 5). These types of conformational changes are common in this protein family, with 11 of the 41 structurally characterized COMT-type methyltransferases displaying unambiguous open and closed conformations (Table S1). Given the abundance of open, closed, and intermediate conformations of COMT-type methyltransferases found in the PDB, it is clear that these conformational changes likely play a role in enzyme catalysis in RedM and related enzymes.

Docking the indolocarbazole substrate into the active site closed conformation highlights several key residues likely important to substrate recognition and catalytic activity. We wished to see if these residues are conserved in the wider COMT-type enzyme family, and whether sequence-based analyses could reveal any determinants of heteroatom methylation or dimethylating activity in general. N-methylating enzymes are found throughout the COMT-type family as a relative minority representing less than 9% of Swiss-Prot annotated sequences. These enzymes methylate a variety of nitrogen functional groups including amines, amides, lactams, anilines, and pyridines. COMT-type enzymes capable of catalyzing multiple methyl transfers have so far only been characterized in plants and bacteria and include RedM, OxyT, RosA, *La*PhzM, BenF, *Cr*OMT2, ROMT, AMNT, and BANMT (Table 2). Checking the substrates methylated by many COMT-type enzymes shows that all contain at least one aromatic ring with example substrates being monolignols (34, 35), hydroxyisoflavones (28, 55), (S)-norcoclaurine (38), and scoulerine (31) in plants, and carminomycin (39), bacteriochlorophyllide (23), tiancimycin (33), and phenazines (24, 30) in bacteria, and some small aromatic substrates such as acetylserotonin in animals (48). The binding cavities of these enzymes isolated in ternary complexes often contain *π*-*π* and S-*π* interactions between residues and the aromatic systems of the bound ligand. It has been observed that methionine residues play a role in stabilizing *π* systems (26, 27). This is represented in RedM where the active site is lined with five methionine residues, with M157, M297, and M301 forming major contacts with the docked indolocarbazole substrate. The first is found in a 153-FxxxM-157 motif in the interdomain helix, which is common throughout plant and bacterial COMT-type enzymes (Fig. S6). In RedM this motif moves to within 4 Å of the methylsulfur moiety of SAM, displacing solvent and likely contacting the indolocarbazole substrate forming a stabilizing edge on *π*-stacking and *π*-sulfur interactions. The opposite face of the substrate is supported by residues 297-MxxxM-301 in RedM. This motif is less widespread, found in 9.1% of sequences in the RedM SSN subcluster; however, generalizing the position 297 to residues that interact favorably with aromatic substrates ([FYML]xxxM) covers 58% of sequences (Fig. 7*C*). These four positions are the active site cavity-facing residues of their respective *α*-helices near the SAM cofactor and are necessarily key interaction points to orient substrates for methyl transfer in the typical COMT-type fold. Sequence conservation in 153-FxxxM-157 suggests the motif is a common strategy, whereas positions 297 and 301 may be more substrate-specific. Overall, no clear residue conservation among N-methyltransferases indicating a preference for nitrogen atoms over oxygen atoms was observed.

**Table 2:** Known COMT-type family enzymes capable of furnishing at least two methyltransfers to at least one substrate

Origin	Name	UniProt	Involved in the biosynthesis of:	Methylated moiety	PDB
bacteria	RedM	A0A0F7G196	reductasporine	aminoalkane	8TJJ
	OxyT (59, 60)	Q3S8P6	oxytetracyclin	aminoalkane	none
	RosA (29)	K4RFM2	roseoflavin	aniline	4D7K
	*La*PhzM (30)	A0A172J1V3	myxin	phenol	6C5B
	BenF (62)	B1GSM8	benastatin	anthrone	none
plant	*Ta*OMT2 (61)	Q38J50	tricetin	phenol	none
	*Cr*OMT2 (45)	Q8GSN1	syringetin	phenol	none
	ROMT (44)	B6VJS4	resveratrol	phenol	none
	AMNT (46)	Q96565	gramine	aminoalkane	none
	BANMT (47)	Q84N56	*β*-alanine betaine	*β*-amine	none

BANMT and TaOMT2 are able to transfer three methyl groups to their base substrates.

While the structure of RedM closely matches that of the COMT-type enzyme family it is missing the characteristic catalytic histidine found in most other members of the family, instead having a serine at this position (S253). Mutation of this serine to alanine or cysteine does not impact catalytic activity, while mutation to histidine eliminates it. Thus, S253 does not play an active role in catalysis, but productive catalysis is sensitive to the steric bulk or side chain charge at this position. The nearby N254 is shown in docking to be in close proximity to the pyrroline nitrogen of the indolocarbazole substrate and may play a role in substrate recognition and orientation for methyl transfer. This is a common proposal among other small molecule methyltransferases with asparagine or aspartate at this position (19, 31, 34, 38, 39).

Mutational studies in COMT-type C-methyltransferase SibL, O-methyltransferase DnrK, and S-methyltransferase DsyB indicate a tyrosine in the two *α*-helices capping the active site between the N- and C-terminal domains affects catalytic activity. In SibL, Y134F eliminates catalytic activity and the residue is proposed to deprotonate the methyl acceptor carbon atom after methylation and reforming an aromatic ring (40). In DnrK, the Y142W mutation reduces catalytic activity by 52% (39), while in DsyB, the Y129F mutation reduces catalytic activity by 60% (41). Each RedM structure has a disordered region around residues 133 to 149, which includes the analogous position (Y140). The Y140F variant has greatly reduced reductasporine accumulation, suggesting the region participates in the completion of the active site and the residue promotes turnover. We speculate that this loop may be transiently stable in order to enable the replacement of SAH with SAM after the first round of methyl transfer. DnrK and DsyB are stated to be “entropic enzymes” where the catalytic rate increase is attributed to desolvation and precise positioning of the substrate (17, 39, 41). RedM may operate under a similar schema given that the presence of tyrosine is not necessary for catalysis unlike the equivalent residue in SibL and no other active site residues are good candidates for direct acid-base chemistry. A similar entropic enzyme explanation is given for the transmethylation mechanism of bacterial N-methyltransferase PhzM, as the enzyme lacks any apparent catalytic base and the low pK_a_ of the phenazine nitrogen is amenable to methyl transfer without extra deprotonation at neutral pH (24).

Searching the literature, Swissprot database and BLAST results suggest that to date nine COMT-type family enzymes have characterized dimethylating activity (Table 2). Multiple sequence alignment and SSN distribution of these does not reveal any clear sequence-based indications of dimethylating activity. The addition of steric bulk to the RedM active site opposite the S-methyl of the SAM cofactor *via* H250W and S253H extinguished methylation activity entirely. H250W may have interfered with effective SAM binding, as H250 interacts with the SAM carboxylate, despite tryptophan being tolerated at this position in several COMT-type enzymes. S253H may be too large of a residue and excludes adequate alignment of the pyrroline nitrogen with SAM. However, this strategy has been effective in altering monomethylating enzymes into dimethylating enzymes and vice-versa. For example, SxtA is a PKS iron-dependent C-methyltransferase structurally similar to MppJ, which methylates malonyl-ACP to methylmalonyl-ACP (56). Mutation of an active site isoleucine to threonine removes steric bulk from the active site and allows for the binding of the monomethylated product in a flipped orientation followed by a second methylation event. The mutation emulates the active site of the closely related dimethylating methyltransferase AprA (57). Due to the warping of the active site in order to accommodate the iron cation and subsequent altered substrate binding, there is no equivalent mutation in RedM. The COMT-S enzyme, responsible for the monomethylation of various hydroxylated coumarins, was converted into a dimethylating enzyme *via* a single mutation N325A (32). N325 is predicted to mediate substrate recognition through hydrogen bonding and the removal of that recognition element combined with the reduction of the side chain size allows for the nonselective methylation of both hydroxyl groups in the substrate esculetin. The equivalent position in RedM is T305, which as a small residue may enable multiple methyl transfers. However, the geometry of the docked indolocarbazole substrate suggests that the substitution of T305 with a larger residue would have little impact on substrate binding. Additionally, other dimethylating COMT-type enzymes have a variety of small (Ala, Thr, Val) and large (Phe, Tyr, Arg, Leu) residues at this position. Lastly, resveratrol O-methyltransferase (ROMT) non-selectively dimethylates resveratrol to pterostilbene, but the mutant L117F, a position far from the site of methyl transfer, is able to primarily accumulate the monomethylated pinostilbene product (58). This demonstrates that subtler manipulations of the binding cavity shape can determine the extent of methylation.

Characterized COMT-type family dimethyltransferases have substrates that are either achiral or highly symmetric. For example, the N-MTs RedM, OxyT (59, 60), RosA (29), AMNT (46), and BANMT (47) each only require an amine inversion or C-N bond rotation for a second methyl transfer to occur. *Ta*OMT2 (61), *Cr*OMT2 (45), and ROMT (44) operate on achiral tricetin, myricetin, and resveratrol, respectively, and require a simple ring flip *via* C-C bond rotation in order to methylate the second phenol position. *Ta*OMT2 is unique in that it can also methylate a third, nonequivalent hydroxyl position in tricetin. *La*PhzM methylates the highly symmetrical iodinin twice and requires a 180° flip of the entire molecule before the second methyl transfer (30). Finally, BenF of the benastatin biosynthetic gene cluster methylates an anthrone carbon position twice (62). The second methyl transfer likely occurs as the planar nucleophilic tautomer of the monomethylated intermediate forms and attacks the SAM cofactor.

Observed multi-methyltransferase activity in COMT-type family enzymes seems to arise from the natural symmetry between nucleophilic positions within a substrate paired with limited or no exclusion of the nearly equivalent monomethylated intermediate from the active site. Notably, the remaining characterized N-methyltransferases each catalyze a single methyl transfer to a nitrogen atom part of a substituted amide or aromatic heterocyclic ring. These are nucleophilic positions that can only support a single methyl group without additional chemical changes. Multi-methylating activity is less tied to specific trends in sequence across methyltransferases and is more dependent on the nature of the substrate and subtle changes in the shape and surface of the active site, as seen in COMT-S, ROMT, AprA, and SxtA. This is also observed in other SAM-dependent enzymes outside the COMT-type family catalyzing sequential methyl transfers, such as glycine/sarcosine N-methyltransferase (63) (GSMT), lysine methyltransferase SET7/9 (64) and rubisco large subunit methyltransferase (65) (LSMT). GSMT is able to catalyze two sequential methylations to glycine to form N-dimethylglycine as part of a biosynthetic pathway to the osmoprotectant betaine (63). When comparing GSMT against the related glycine N-methyltransferase (29% identity; RMSD = 2.1 Å, 230 C_*α*_), which only catalyzes a single methyl transfer to glycine, the active site pocket has a marked smaller volume incapable of accommodating N-methylglycine in a conformation for productive methylation. Similarly, the active site mutations Y305F and Y245A in SET7/9 expand accessibility to methylated substrates shifting catalytic activity from monomethylation to dimethylation or trimethylation, respectively (64). LSMT forms a more open active site compared to the similar enzyme SET7/9, and co-crystal structures with N_***ε***_-methyl-l-lysine show that the N-methyl preferentially points away from the SAM cofactor leaving an unobstructed path for further methylation events (65).

Furthermore, the ability of the enzyme to cycle between open and closed conformations easily would allow for the substrate and SAM to be captured in the open conformation, transmethylated upon closure of the enzyme, followed by release of SAH and replacement with SAM in the open conformation. Potentially, the singly methylated intermediate can be retained in the open active site where the necessary rotations or inversions can occur before a second methylation with a freshly acquired SAM cofactor. Docking the indolocarbazole substrate to the open conformation of RedM seems to indicate a preference for binding far away from the SAM binding site near *α*1, *α*2, and the loop between *β*9 and *β*10 (Fig. 5*A*). The indolocarbazole substrate may access the active site cavity from this position and be temporarily held there between methyltransfers. However, mutating the position immediately between *α*1 and *α*2 (T22Q) in order to disrupt any such interaction does not alter catalytic activity noticeably. In *La*PhzM, this end of the active site is proposed to control substrate access to the enzyme, where the residues Y158 and F290 form a junction limiting the maximal size of a molecule able to access the active site (30). L160 and M297 in RedM form a similar gatekeeping junction where upon closure of the enzyme they contact the other forming a ‘floor’ to the active site.

The N,N-dimethyltransferase RedM of the reductasporine biosynthetic pathway is the third example of a COMT-type family N-methyltransferase to have its structure characterized and the first to be captured in open and closed conformations. Docking didemethylreductasporine to the closed conformation indicated key amino acid residues in substrate recognition. Sequence analysis shows that these residues are widely conserved as an FxxxM motif in the *α*-helix joining the N- and C-terminal domains and a reciprocal [FYML]xxxM motif on the opposite wall of the active site. The second motif is less strictly conserved but is preferentially found as large hydrophobic residues. Mutations to the active site indicate no residues are likely to participate directly in catalysis, suggesting RedM operates as an entropic enzyme leveraging desolvation, orientation, and proximity effects to promote methyl transfer, as seen in other COMT-type family enzymes (24, 39, 41). Finally, it seems that dimethylating activity in COMT-type enzymes is a natural result of processing largely symmetric, planar substrates where the enzyme cannot distinguish between nearly identical positions after amine inversions, bond rotation, or tautomerization. These crystal structures give insight into the final steps of reductasporine biosynthesis and highlight the importance of domain movements in catalysis for this family of enzymes.

## Experimental procedures

### General methods

Primers were synthesized by Integrated DNA Technologies. Q5 DNA polymerase, T4 DNA ligase, and restriction endonucleases were purchased from New England Biolabs. DNA sequencing was carried out by either NAPS Unit DNA Sequencing Facility or CMMT/BCCHR DNA Sequencing Core Facility (University the British Columbia). Nickel sepharose protein purification resin and HiLoad 26/60 Superdex 200pg size exclusion column was purchased from GE Healthcare. ISP4 and TSB media were purchased from BD. Other general reagents were purchased from Anatrace, Bio Basic Inc, Hampton Research, Sigma-Aldrich, Thermo Fisher Scientific Canada, and VWR International as necessary. HPLC analysis was carried out on an Agilent 1260 HPLC apparatus, and LC-MS analysis on an Agilent 6120 Quadruple LC/MS system in positive and negative ion mode.

### Cloning and expression of redE and redM

The gene *redE* was assembled from 36 oligonucleotides designed to exclude NdeI and XhoI restriction sites using the DNAWorks software (66). The gene *redM* was codon optimized and cloned into pUC17 by Bio Basic Incorporated. Both *redE* and *redM* were amplified and ligated into the NdeI/XhoI sites of the pET28a plasmid.

Plasmids pET28a-*redE* or pET28a-*redM* were transformed into chemically competent *E. coli* BL21(DE3) and grown in lysogen broth (LB; 10 g NaCl, 10 g tryptone, 5 g yeast extract per 1 L water) (37 °C, 180 rpm) with kanamycin (50 *μ*g/ml) to an OD_600_ of 0.6 before cooling to 16 °C (30 min). Cultures were induced with 100 *μ*M β-d-1-thiogalactopyranoside (IPTG) and grown for a further 18 h.

Cells were harvested by centrifugation (4 °C, 10 min, 3000*g*), and resuspended in 50 mM Tris (pH 8), 500 mM NaCl, 5 mM imidazole and 1.5 mM tris(2-carboxyethyl)phosphine (TCEP). Bacteria were lysed by sonication (25% amplitude, 4 s on/8 s off cycles, 6 min), and cell debris was removed *via* centrifugation (45 min, 15,000*g*). The supernatant was loaded onto Ni-IDA affinity resin and then eluted using a 20 to 500 mM imidazole step gradient in 50 mM Tris (pH 8), 500 mM NaCl, 1.5 mM TCEP. Elution fractions containing recombinant protein were filtered through a 0.22 *μ*m syringe filter and purified *via* size-exclusion chromatography in 20 mM Tris (pH 8), 50 mM NaCl, and 1.5 mM TCEP. SDS-PAGE was used to assess the purity of resulting fractions.

### Cloning and expression of rebO and rebD, and production of chromopyrrolic acid

The genes *rebO* and *rebD* were amplified from gDNA (*Lechevalieria aerocolonigenes* DSM 44217). These were ligated into pET28a and pET22b, respectively. *E. coli* BL21(DE3) cells overexpressing RebO and RebD were grown according to the previously published procedure (6).

Cells were harvested by centrifugation (4 °C, 10 min, 3000*g*), and resuspended in 20 mM Tris (pH 8), 500 mM NaCl, 5 mM imidazole. Bacteria were lysed by sonication (25% amplitude, cycles of 4 s on and 8 s off for 6 min), and cell debris was removed *via* centrifugation (45 min, 15,000*g*). The cleared supernatants containing RebO and RebD were added to a solution of l-Trp (100 mg, 490 *μ*mol) buffered in 25 mM HEPES (pH 8, 250 ml) and stirred for 18 h at room temperature. The reaction was quenched *via* the addition of HCl until pH <2 before removing the precipitate *via* centrifugation. The supernatant was passed through a Waters 20 cc C18 Sep-Pak cartridge and eluted by a H_2_O: MeOH step gradient (0–100% MeOH). The fractions containing chromopyrrolic acid were combined, concentrated, and purified using a semi-preparative C18 column (Phenomenex Luna C18(2), 100 Å, 250 × 10 mm, 5 *μ*m) 5 to 100% ACN in H_2_O.

### Expression and purification of StaP, ferredoxin, and ferredoxin reductase

Plasmid pET28a-*staP* was kindly provided by Catherine Drennan (Massachusetts Institute of Technology), and plasmid stocks of ferredoxin-NADP oxidoreductase (FDR) and ferredoxin I (FDX) from *S. elongatus* PCC 7942 were received from Dr Hai-Yan He.

*E. coli* BL21(DE3) harboring pET28a-*staP* or pET28a-*fdx* were grown in LB medium with kanamycin (50 *μ*g/ml) to OD_600_ of 0.5 before moving to 16 °C for 30 min 1 mM *δ*-aminolevulinic acid and 100 *μ*M IPTG were added to cultures before growing for a further 18 h. *E. coli* BL21(DE3) transformed with pET28a-*fdr* were grown similarly with the omission of *δ*-aminolevulinic acid.

Cells were harvested by centrifugation (4 °C, 10 min, 3000*g*) and resuspended in 50 mM Tris (pH 8), 500 mM NaCl, and 5 mM imidazole. Bacteria were lysed *via* sonication (25% amplitude, cycles of 4 s on and 8 s off for 6 min), and cleared *via* centrifugation (45 min, 15,000*g*). Soluble 6×His tagged StaP, FDX, and FDR were purified *via* Ni affinity column and imidazole step gradient (5–300 mM). Dialyzed protein fractions were screened for activity against CPA before adding glycerol to 10% (v/v) and storing at −70 °C.

### *In vitro* production of reductasporine and pathway intermediates

Dicarboxyindolocarbazole was generated *in situ* from CPA and StaP in a tandem reaction containing 250 *μ*M CPA, 5 mM NADPH, 50 mM HEPES pH 8, 1 *μ*M StaP, 20 *μ*M FDX, 1 *μ*M FDR and 20 *μ*M RedE. Reductasporine production used the same conditions supplemented with 5 mM SAM and 20 *μ*M RedM. Reactions were quenched with two volumes of MeOH and denatured protein was removed by centrifugation. HPLC assays were run on an Agilent 1260 HPLC apparatus with a Phenomenex Luna C18(2), 100 Å, 250 × 4.6 mm, 5 *μ*m column at 1 ml min^−1^ using a gradient of 2 to 100% ACN in 0.1% formic acid. Elution was monitored at 260, 280, 300, and 325 nm or by mass.

Large-scale preparation of reductasporine used the same conditions as above at a total volume of 3 ml and the reaction was supplemented with extra NADPH, StaP, FDX, and FDR after two and 4 h. ^1^H-NMR (600 MHz, DMSO-d6) δ 3.53 (6H, s, H_3_-14, H_3_-14a), 5.52 (4H, s, H_2_-5, H_2_-7), 7.29 (2H, t, H-3, H-9), 7.48 (2H, t, H-2, H-10), 7.78 (2H, d, H-1, H-11), 7.96 (2H, d, H-4, H-8), 11.43 (2H, brs, NH-12, NH-13)

### Synthesis of arcyriarubin A

Based on procedures published by Faul *et al.* 1995 and Mei *et al.* 2017 (67, 68). To a N_2_ purged suspension of 52 mg magnesium (2.1 mmol) in 2 ml THF, 170 *μ*l of ethyl bromide (2.3 mmol) was added and stirred at 40 °C for 1 h. Indole solution (260 mg, 2.2 mmol, 2 ml toluene) was added dropwise before heating at 60 °C for 1 h. Dibromomaleimide (78 mg, 0.31 mmol, 1 ml THF) was added slowly before refluxing for 15 h. Quench proceeded with 1 M HCl and was partitioned into ethyl acetate and water. Organic crude was washed with water and brine and the aqueous wash was extracted with ethyl acetate. The combined organic phase was dried with sodium sulfate and solvent was removed. The crude was purified *via* silica column (1:1 hexanes:ethyl acetate), yielding 50 mg of arcyriarubin A as a red oil (50% yield).

### Synthesis of arcyriaflavin A

Based on the procedure published by Reddy *et al.* 2003 (69). Iodine (8 mg, 32 *μ*mol) was added to an arcyriarubin A solution (50 mg, 150 *μ*mol, 24 ml 1:1 THF:ACN), and stirred under a mercury lamp (400 W) for 23 h. Solvent was removed and dissolved in 2 ml THF before precipitating product with 15 to 20 ml hexane. The filtered solids were dried under vacuum and used without further purification (45 mg, 91% yield).

### Synthesis of didemethylreductasporine (3)

Based on previously published amide (70), lactam (71) and isoindoline-1,3-dione reductions (72, 73). A suspension of NaBH_4_ in THF (28 mg, 0.5 ml) was purged with N_2_, before adding arcyriaflavin A (10 mg, 31 *μ*mol, 1 ml THF). The solution was cooled to 0 °C and 250 *μ*l of a 370 mM iodine solution in THF was added dropwise over 10 min. The reaction was stirred for 2.5 h before bringing it to 65 °C for 16 h. Quench proceeded with 400 *μ*l methanol followed by 1 M HCl and partitioning between ethyl acetate and water before washing with 1 M NaOH and brine. Semi-preparative HPLC at 30% ACN yields didemethylreductasporine (5 mg, 50% yield) with K252c identified as the major side product alongside unreacted arcyriaflavin A.

### Enzymatic methylation of didemethylreductasporine

RedM activity was assayed independently from RedE by including 2 *μ*M RedM, 2 mM SAM, 500 *μ*M didemethylreductasporine, 50 mM HEPES pH 8 in a reaction mixture. Aliquots of the reaction mixture were taken at 5, 10 15, 20, and 25 min and quenched with two volumes of acetonitrile. Reaction products were measured *via* HPLC on a Phenomenex Luna C18(2), 100 Å, 250 × 4.6 mm, 5 *μ*m column at 0.8 ml min^−1^ using a gradient of 5 to 90% ACN in 0.1% formic acid over 11 min. Elution was monitored by UV-Vis absorbance at 325 nm and by mass.

### Selenomethionine incorporation into RedM

Plasmid pET28a(+)-*redM* was transformed into *E. coli* BL21(DE3) expression hosts and plated on kanamycin-supplemented LB agar for overnight growth. Single colonies were grown for 8 h in 5 ml LB with kanamycin before inoculating 30 μl into 30 ml of M9 minimal medium and grown overnight. This starter culture (10 ml) was added to 1 L of M9 medium with kanamycin and grown to an OD_600_ of 0.7 AU before cooling to 16 °C l-configured lysine, threonine, and phenylalanine (100 mg each), and leucine, isoleucine, valine, and selenomethionine (50 mg each) were combined and added to each 1 L culture (74). After 25 min of mixing, cultures were induced with 100 μM IPTG and grown for 16 to 18 h. Selenomethionine RedM derivatives were purified as described previously with buffers freshly supplemented with 5 mM β-mercaptoethanol.

### Crystallization of RedM

Initial conditions amenable to RedM crystallization were determined *via* commercially available trial screens (Index, Hampton, MCSG) in a 96-well plate, sitting-drop format (1 μl protein: 1 μl crystallization solution adjacent to 50 μl of mother liquor). In 0.2 M (NH_4_)_2_SO_4_, 0.1 M MES pH 6.5, 30% w/v polyethylene glycol monomethylether 5000, RedM at 7 mg/ml crystallized as plates within 3 weeks or in 0.05 M (NH_4_)_2_SO_4_, 0.05 M BisTris-HCl pH 6.5, pentaerythritol ethoxylate (15/4_EO/OH) at 17 mg/ml RedM as prisms. Initial hits were optimized in hanging-drop screens set up in 24-well plates with drops of 1 to 2 μl of protein (13–30 mg/ml) and 1 to 2 μl crystallization solution.

The optimal growth of RedM selenomethionine derivative crystals was found to be 0.3 M (NH_4_)_2_SO_4_, 0.1 M Tris pH 8.0, 30% PEG MME 5000 at 12.5 mg/ml protein. Diffraction quality native RedM crystals grew under 0.2 to 0.3 M (NH_4_)_2_SO_4_, 0.1 M MES pH 6.5, 18 to 24% polyethyleneglycol monomethyl ether 5000 at 13 to 16 mg/ml RedM, as well as 0.09 M (NH_4_)_2_SO_4_, 0.05 M BisTris-HCl pH 6.5, 30% pentaerythritol ethoxylate (15/4_EO/OH) at 30 mg/ml RedM.

The RedM-SAH crystal was retrieved from a pentaerythritol ethoxylate condition before soaking for 3 h in freshly made well solution supplemented with 1 mM S-adenosylhomocysteine. This crystal was moved to a cryoprotectant solution consisting of the well solution made with 10% (v/v) ethylene glycol for less than 30 s before flash freezing under a gaseous stream of nitrogen held at 100 K. The RedM-SAM crystal originated from a polyethylene glycol condition and was soaked in freshly mixed well solution supplemented with 10 mM S-adenosylmethionine for 5 min. Unliganded RedM and RedM-SAM crystals were moved into cryoprotectants consisting of the mother liquor and 20% (v/v) ethylene glycol for less than 30 s, before flash-freezing in nitrogen. These crystals were tested locally for diffraction using a Rigaku MicroMax-007HF (1.54 Å, rotating copper anode) with Saturn 944+ CCD detector before storing for further data collection at a synchrotron facility.

### Data collection, structure determination, and refinement

Datasets for RedM were collected at the Canadian Light Source (CLS, Saskatoon, Canada) on beamline 08B1-1 (Saskatoon, Canada) using a Rayonix MX300-HE detector, and at the Stanford Synchrotron Radiation Lightsource (SSRL, Menlo Park, United States) on beamline BL9-2 with a Pilatus 6M PAD detector. Diffraction images were processed and integrated using iMOSFLM (75, 76) or XDS (77). Data were scaled and merged in AIMLESS (78), and phases were determined by single anomalous dispersion of selenium atoms or *via* molecular replacement of the partially solved selenomethionine-derived RedM model using the PHENIX software package (79). An initial model was created and refined using the PHENIX Autobuild software (80). This initial model was improved upon through cycles of manual modelling with Coot (81) and refinement with REFMAC (82) and phenix.refine (83).

### Substrate docking

Molecular docking of didemethylreductasporine, demethylreductasporine, and reductasporine with open and closed conformations of RedM was performed using AutoDock VINA (84). Coordinates of K252c were retrieved from the Protein Data Bank (PDB ligand code: K2C) and modified in PyMOL (85) to each reductasporine derivative before optimizing geometries using eLBOW (86). RedM protein models bound with SAM and SAH were prepared for docking by removing water, adding exchangeable hydrogen atoms, and designating flexible residues *via* MGLTools for AutoDock. Docking was bound to a 40 × 40 × 40 Å box centered in the cavity adjacent to SAH or SAM and contained the majority of the interior surface of both open and closed conformations of the RedM monomer. The exhaustiveness parameter was set to 24 after no noticeable changes to docking outcomes were observed at higher values. Analysis and visualization of docking results were done using PyMOL (85).

### Site-directed mutagenesis of RedM

Primers were designed with an overlapping region containing the mutation and 5′ non-overlapping arms. Overlapping areas were chosen with melting temperatures of approximately 55 °C and non-overlapping regions had melting temperatures about 7 °C higher (calculated against the complementary DNA strand) (87). Standard PCR was carried out using the pET28a(+)-*redM* plasmid with an annealing step set to the higher melting temperature and ended with a 10 min elongation step. The PCR product mixture was incubated with DpnI before transforming the digest solution as described previously into *E. coli* DH5*α*. Mutations were confirmed by sequencing.

### Visualization and analysis software

Crystal structures are visualized using Pymol (85). Estimates of internal cavity volume were generated with POCASA (grid size = 0.5 Å, probe radius = 3 Å, single point flag = 11, protein depth flag = 10) (25). Surface areas and dimer interaction surfaces were calculated *via* PISA (14). Bending residues and hinge axes were found through DynDom (88). Protein sequence alignments were generated using Clustal Omega (89) or COBALT (90) and visualized with Jalview (91).

### Sequence similarity networks

Initial SSN was created using the EFI-EST service (92) for the two annotated dimerization domains (plant methyltransferase dimerization, IPR012967; acetylserotonin O-methyltransferase dimerization, IPR031725); however, the network covered approximately half of the known structural homologs to RedM. The query was expanded to include the associated methyltransferase domain (IPR001077) while reducing the granularity of sequence space by using the UniRef50 database. The resulting network was missing only two RedM structural homologs from the 41 represented in the PDB (MppJ and Rv2258c). Edges were removed incrementally until distinct clusters formed between enzymes from plants, animals/fungus, and bacteria (an alignment score >54). All sequences from the cluster containing RedM were retrieved and reanalyzed *via* EFI-EST for a more granular SSN of related enzymes. The EFI-GNT tool was used to look up local gene arrangements in order to identify candidate biosynthetic gene clusters as an indication of isofunctionality within a cluster.

## Data availability

Structures of unliganded RedM, RedM-SAM and RedM-SAH have been deposited to the Protein Data Bank under PDB IDs 8TJI, 8TJJ and 8TJK.

## Supporting information

This article contains supporting information (19, 31, 35, 38, 40, 41, 42, 90, 93, 94).

## Conflict of interest

The authors declare that they have no known competing financial interests or personal relationships that could have appeared to influence the work reported in this paper.
