# Supplementary material for: Structure of methyltransferase RedM that forms the dimethylpyrrolinium of the bisindole reductasporine
Source: J Biol Chem. 2023 Nov 30;300(1):105520. doi: 10.1016/j.jbc.2023.105520 (PMC10784701; doi:10.1016/j.jbc.2023.105520)
Supplement: Supporting Table S1 and Figures S1–S7 [file mmc1.docx]

Supporting Information for

**Structure of methyltransferase RedM that forms the dimethylpyrrolinium of the bisindole reductasporine**

Phillip Daniel-Ivad^1^ and Katherine S. Ryan^1,^*

Department of Chemistry, The University of British Columbia, Vancouver, Canada

* For correspondence: Katherine S. Ryan, ksryan@chem.ubc.ca

[**Table S1** – COMT-type family methyltransferases with distinct open and closed conformations *2*](#TableS1)

[**Figure S1** – Representative SDS-PAGE gels of RedE and RedM purifications *3*](#FigureS1)

[**Figure S2** – ^1^H-NMR of reductasporine *4*](#FigureS2)

[**Figure S3** – Size-exclusion chromatography traces of RedM and molecular weight standards *5*](#FigureS3)

[**Figure S4** – Chain by chain superpositions highlighting Rossmann-like domain movement *6*](#FigureS4)

[**Figure S5** – Displacements of Rossman-like domain C_𝛼_ centres of mass *7*](#FigureS5)

[**Figure S6** – Sequence alignment of RedM against representative structural homologues *8*](#FigureS6)

[**Figure S7** – View of the crystal packing of unliganded RedM and the RedM-SAM binary complex *9*](#FigureS7)

[**References** *10*](#References)

**Table S1.** COMT-type methyltransferase family enzymes with distinct open and closed conformations. The COMT-type methyltransferase family is characterized with an N-terminal 𝛼-helical dimerization bundle and C-terminal Rossman-like domain which binds an S-adenosyl methionine cofactor.

| **Name** | **Source** | **Function** | **Open Conformations** | **Closed Conformations** |
| --- | --- | --- | --- | --- |
|  |  |  |  |  |
| RdmB^1^ | *Streptomyces purpurascens* | aclacinomycin 10-hydroxylase | 1xdu 1qzz 1r00 | 1xds |
| NcsB1^2^ | *Streptomyces carzinostaticus* | neocarzinostatin O-methyltransferase | 3i58 3i5u 3i64 | 3i53 |
| *Lp*COMT^3^ | *Lolium perenne* (perennial ryegrass) | caffeic acid O-methyltransferase | 3p9c | 3p9i 3p9k |
| MppJ^4^ | *Streptomyces hygroscopicus* | phenylpyruvate C(3)-methyltransferase | 4kif 4kig | 4kib 4kic |
| SibL^5^ | *Streptosporangium sibiricum* | 3-hydroxykynurenine  C-methyltransferase | 4qvg | 4x3q 4u1q |
| 6OMT^6^ | *Thalictrum flavum* subsp*. glaucum* (Yellow meadow rue) | (RS)-norcoclaurine  6-O-methyltransferase | 5icg | 5icc 5ice 5icf |
| *Ps*SOMT1^7^ | *Papaver somniferum* (Opium poppy) | scoulerine 9-O-methyltransferase 1 | 6i5q 6i5z | 6i6k 6i6l 6i6m 6i6n |
| *Fa*OMT1^a^ | *Fragaria ananassa* (Strawberry) | furaneol O-methyltransferase | 6i70 | 6i71 6yjw 6i72 6i73 |
| *Tf*SOMT^a^ | *Thalictrum flavum* subsp*. glaucum* (Yellow meadow rue) | (S)-scoulerine 9-O-methyltransferase | 6nei | 6nej |
| PigF^8^ | *Serratia marcescens* | 4-hydroxy-2,20-bipyrrole-5-carbaldehyde O-methyltransferase | 7clu | 7clf |
| DsyB^9^ | *Nisaea denitrificans* | 4-methylthio-2-hydroxybutyrate  S-methyltransferase | 7wdq | 7wdw |

^a^ structure deposited to the PDB


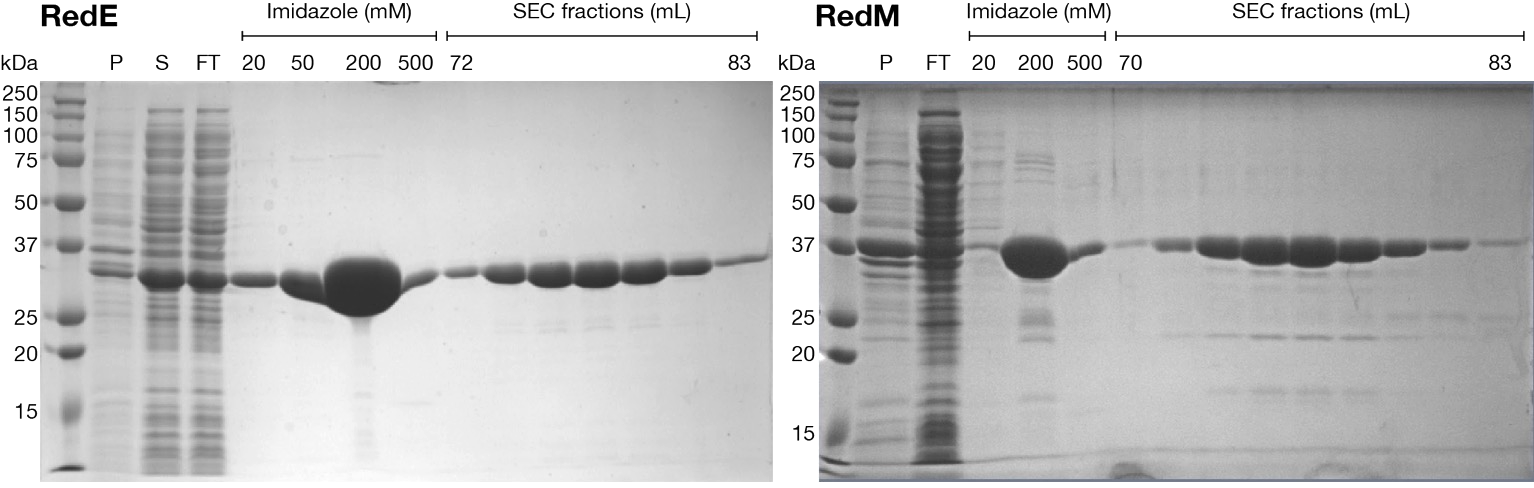


**Figure S1.** Representative SDS-PAGE gels of recombinant RedE and RedM Ni-IMAC and size-exclusion chromatography fractions. P, cell debris pellet; S, supernatant; FT, column flow-through.

**
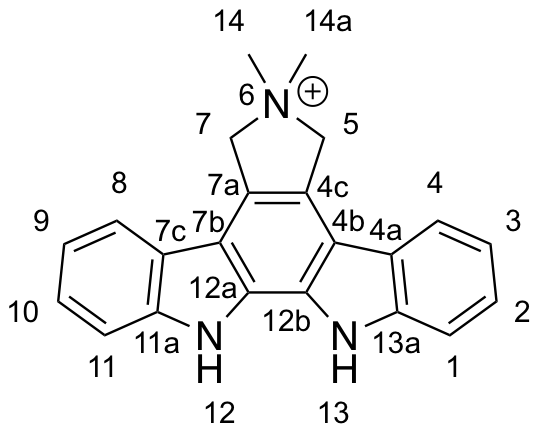
**

**Figure S2.** ^1^H-NMR (600 MHz, DMSO-*d*_6_) of reductasporine isolated from a large-scale in vitro reconstitution of biosynthetic pathway from CPA.


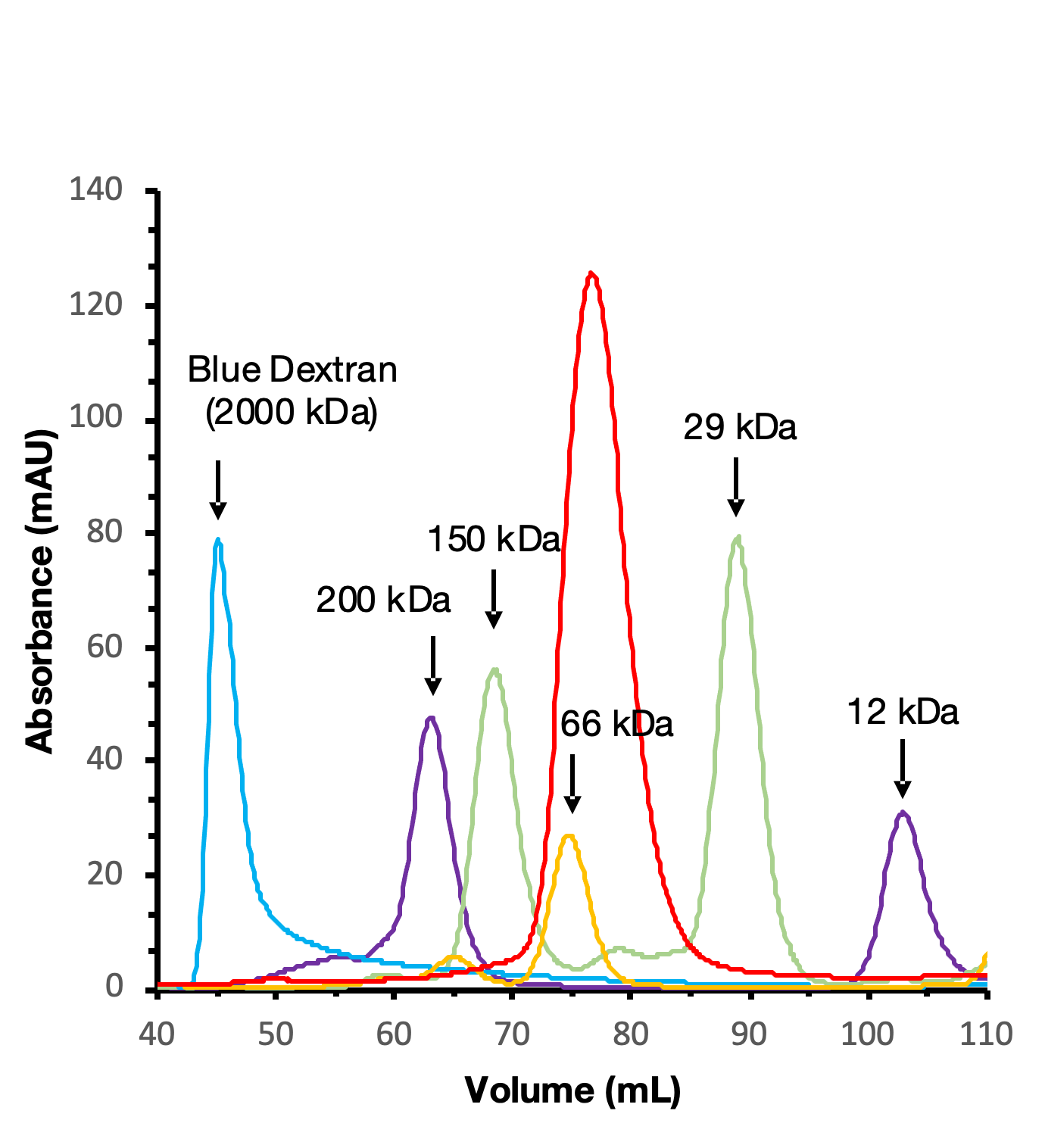


**Figure S3.** Size-exclusion chromatography traces of absorbance at 280 nm for RedM (red) and molecular weight standards. (RedM dimer, 76 kDa; RedM monomer, 38 kDa; sweet potato 𝛽-amylase, 200 kDa; yeast alcohol dehydrogenase, 150 kDa; bovine serum albumin, 66.5 kDa; bovine carbonic anhydrase, 29 kDa; equine cytochrome c, 12 kDa)


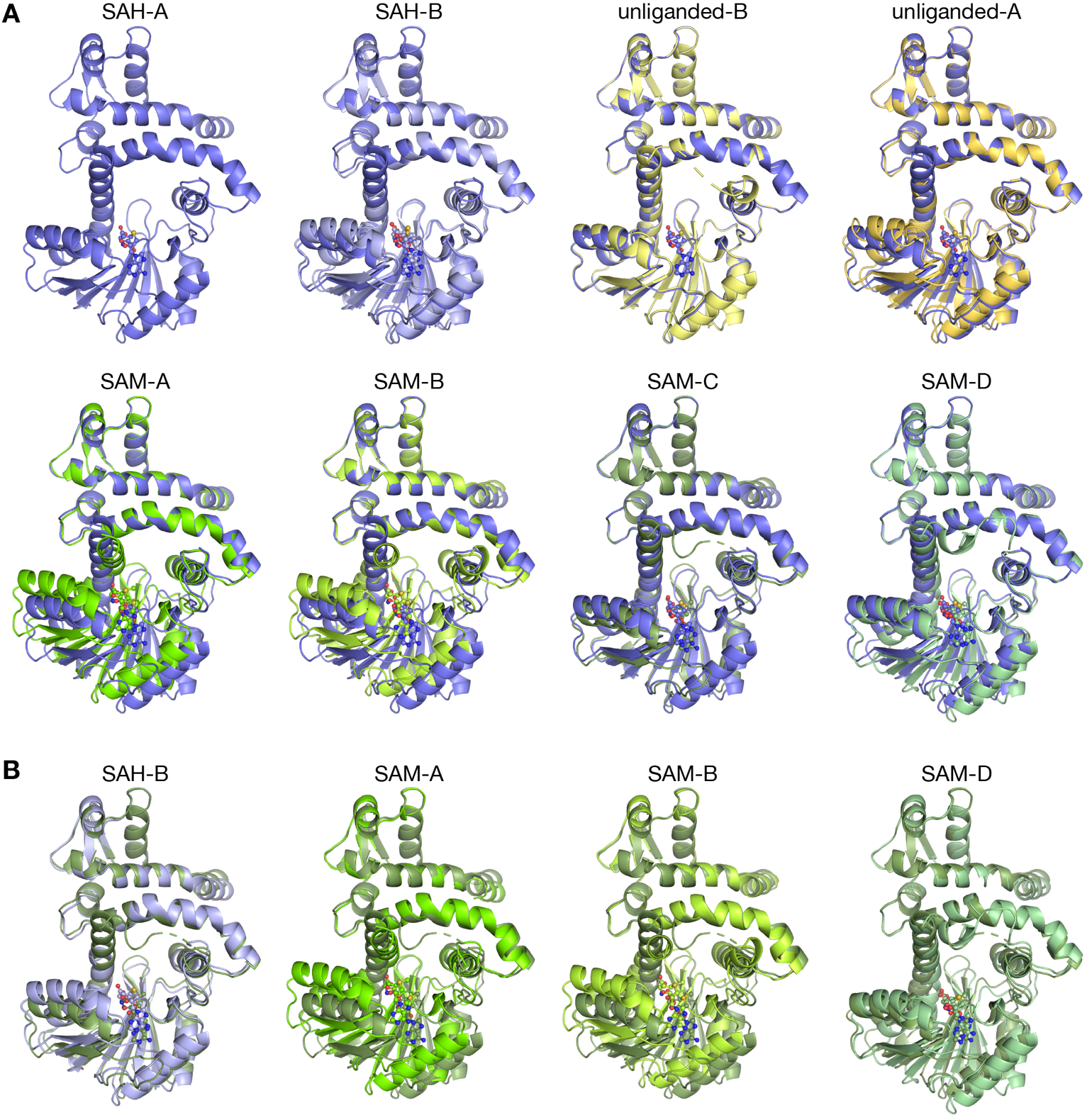


**Figure S4.** Chain by chain superpositions between each protomer of the unliganded-RedM, RedM-SAH and RedM-SAM models and (A) RedM-SAH chain A, dark blue or (B) RedM-SAM chain C, dark green. Each chain is superposed per their N-terminal dimerization domain to emphasize the relative movement of the C-terminal Rossmann-like domain. Chains A and B of the RedM-SAM complex are distinctly closed conformations with markedly reduced internal cavity volumes, while other chains adopt more open conformations. Chain C of the RedM-SAM complex has a more exaggerated open conformation compared to these others, while chain B of the RedM-SAH complex adopts a slightly more closed conformation compared to its dimer partner, chain A.


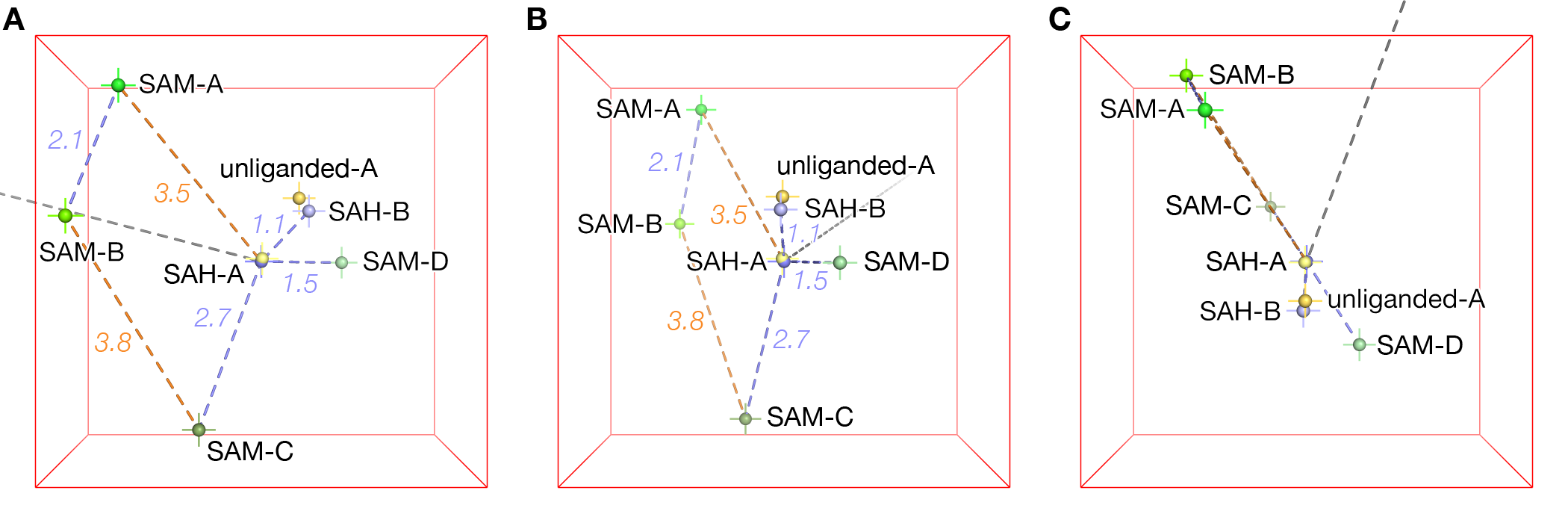


**Figure S5.** Locations of Rossmann-like domain C_𝛼_ centre of mass per chain for each modelled RedM complex viewed along the (A) X-, (B) Y- and (C) Z-axis highlighting the range of movement captured via crystallography. Each chain is aligned per their N-terminal dimerization domains and a grey dashed line indicates the direction towards their centres of mass. Chains A and B of the RedM-SAM complex take on closed conformations with reduced internal cavity volumes, while the others have more open conformations. The transitions from SAH-A to SAM-A and SAM-C to SAM-B rotate about an axis roughly aligned with 𝛼15, while transitions from SAH-A to SAM-C and SAM-A to SAM-B rotate about an axis parallel to helix 𝛼8. The centre of mass of the C-terminal domain corresponds to the position of G249, which interacts with the methionine moiety of the cofactor, approximating the relative movement of the cofactor between conformations. Displacements between centres of mass of like conformations are indicated by blue dashed lines and those of dissimilar conformations by orange dashed lines. A 6×6×6 Å cube is centred on the centre of mass of chain A from the RedM-SAH complex. The direction to the centre of mass of the N-terminal domain is indicated by a grey dashed line. The centre of mass for chain B of the unliganded RedM model (pale yellow) overlaps that of chain A of the RedM-SAH complex and is left unlabelled. All distances are in Angstroms.

**
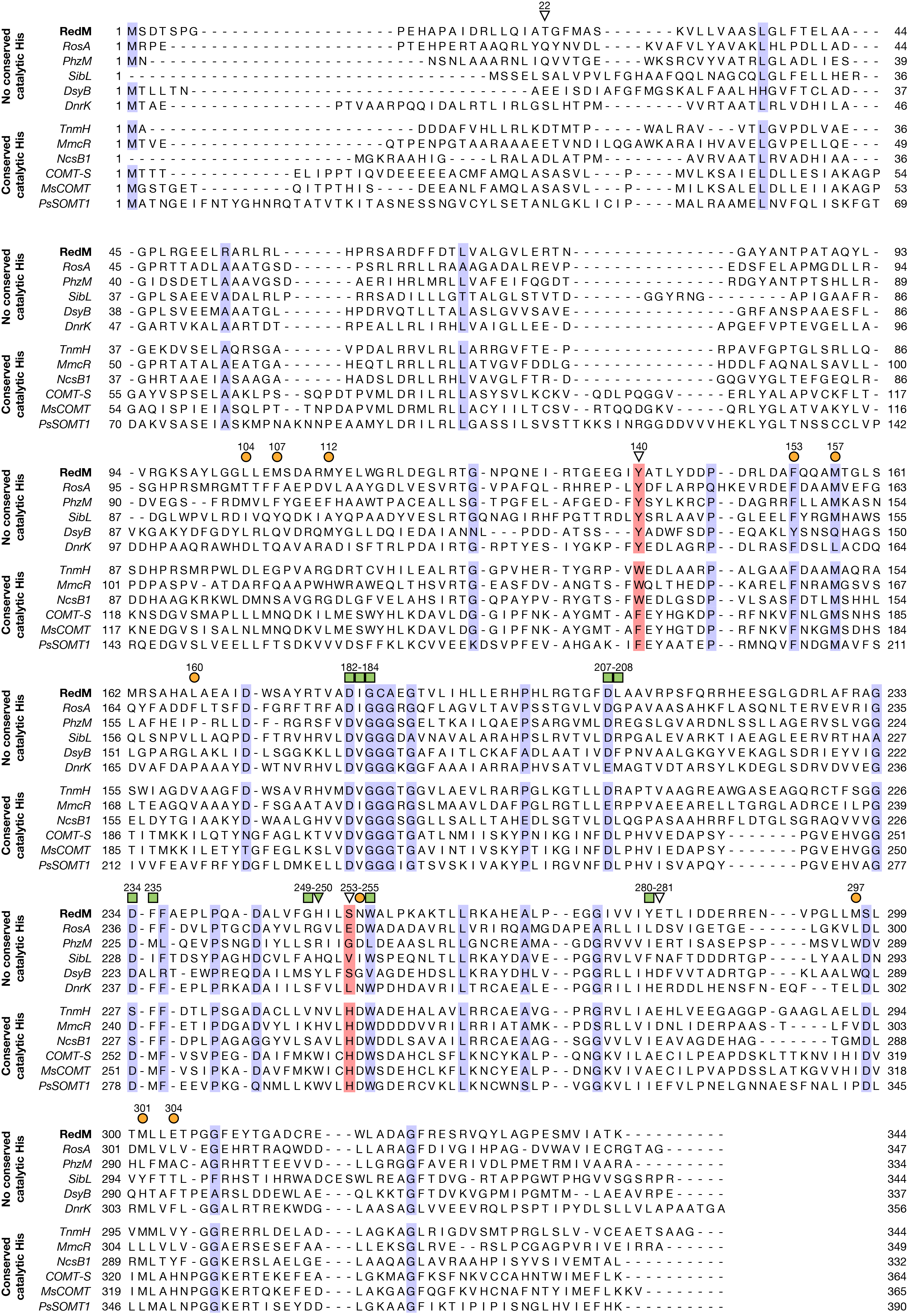
**

**Figure S6.** Sequence alignment of RedM against representative structural homologues available in the PDB grouped by presence or lack of the catalytic histidine found as serine at position 253 in RedM. Residues conserved above 80 % identity are highlighted blue, and positions of catalytically important positions highlighted in pink. RedM substrate binding residues are marked with orange circles, SAM-binding residues with green squares, and residues mutated in RedM with arrows. Alignment was generated using COBALT.^10^ UniProt accessions numbers: RedM (A0A0F7G196), RosA (K4RFM2), PhzM (Q9HWH2), SibL (C0LTM6), DnrK (Q06528), TnmH (A0A125SA05), MmcR (Q9X5T6), NcsB1 (Q84HC8), COMT-S (A0A4P8DY91), MsCOMT (P28002), PsSOMT1 (I3V6A7). NCBI accession numbers: DsyB (WP_028467200.1).

| 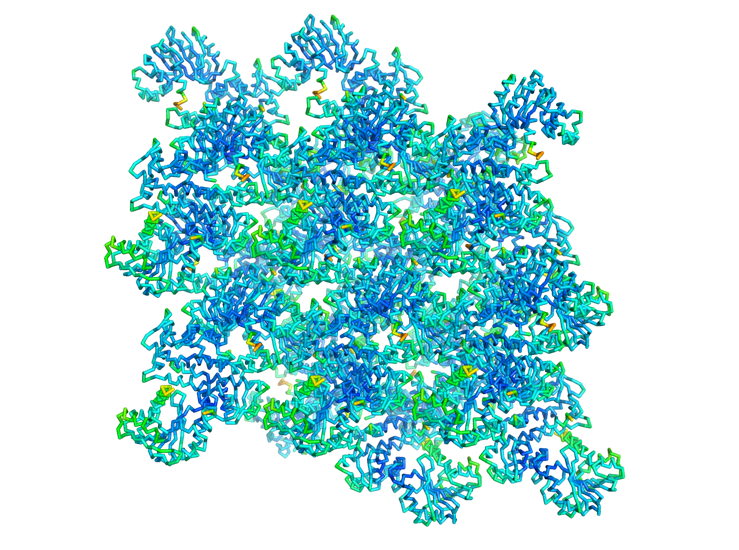 | 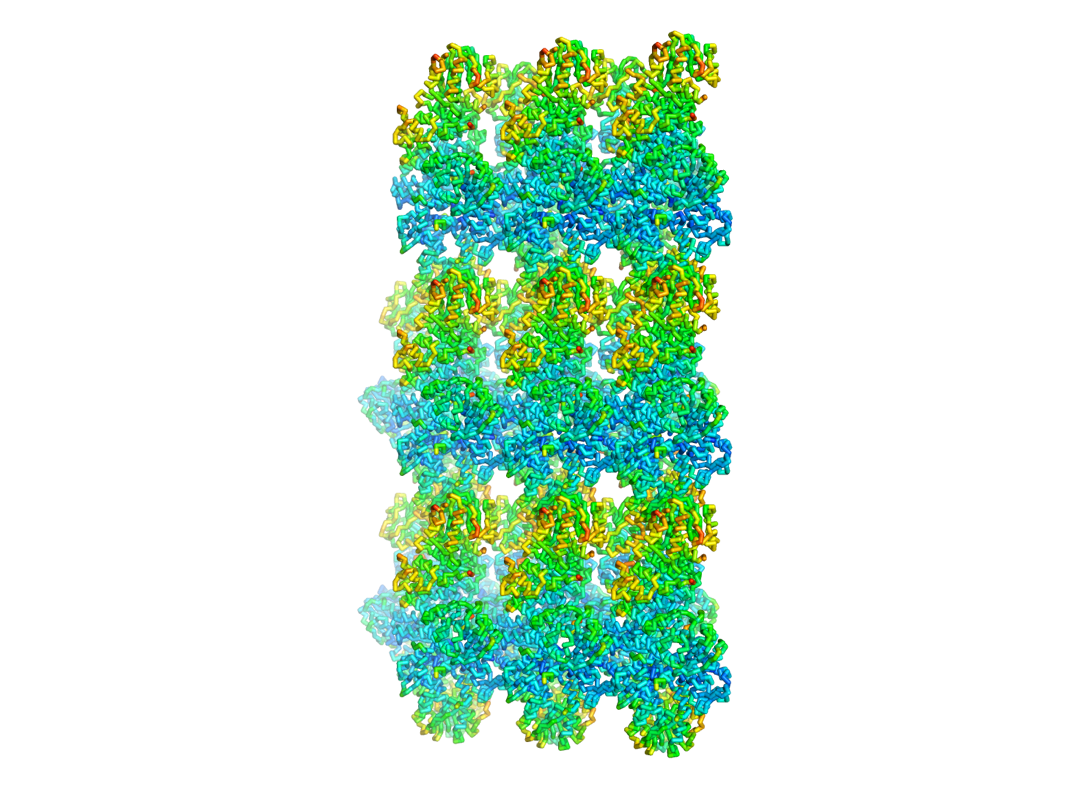 |
| --- | --- |
| Unliganded RedM | RedM-SAM |

**Figure S7.** View of the crystal packing of unliganded RedM and the RedM-SAM binary complex displayed as C_𝛼_ traces and qualitative coloring by B-factor from blue to green to red. The unliganded RedM structure shows low average B-factors with high values only seen in less organized loop regions. The RedM-SAM binary model shows that the crystal forms planes of well packed open conformation chains with low B-factors alternating with poorly packed closed conformation chains with higher B-factors.

**References**

(1) Jansson, A.; Koskiniemi, H.; Erola, A.; Wang, J.; Mäntsälä, P.; Schneider, G.; Niemi, J. Aclacinomycin 10-Hydroxylase Is a Novel Substrate-Assisted Hydroxylase Requiring S-Adenosyl-ʟ-Methionine as Cofactor. *J. Biol. Chem.* **2005**, *280* (5), 3636–3644. https://doi.org/10.1074/jbc.M412095200.

(2) Cooke, H. A.; Guenther, E. L.; Luo, Y.; Shen, B.; Bruner, S. D. Molecular Basis of Substrate Promiscuity for the SAM-Dependent O-Methyltransferase NcsB1, Involved in the Biosynthesis of the Enediyne Antitumor Antibiotic Neocarzinostatin. *Biochemistry* **2009**, *48* (40), 9590–9598. https://doi.org/10.1021/bi901257q.

(3) Louie, G. V.; Bowman, M. E.; Tu, Y.; Mouradov, A.; Spangenberg, G.; Noel, J. P. Structure-Function Analyses of a Caffeic Acid O-Methyltransferase from Perennial Ryegrass Reveal the Molecular Basis for Substrate Preference. *Plant Cell* **2010**, *22* (12), 4114–4127. https://doi.org/10.1105/tpc.110.077578.

(4) Zou, X.-W.; Liu, Y.-C.; Hsu, N.-S.; Huang, C.-J.; Lyu, S.-Y.; Chan, H.-C.; Chang, C.-Y.; Yeh, H.-W.; Lin, K.-H.; Wu, C.-J.; Tsai, M.-D.; Li, T.-L. Structure and Mechanism of a Nonhaem-Iron SAM-Dependent C-Methyltransferase and Its Engineering to a Hydratase and an O-Methyltransferase. *Acta Crystallogr. D Biol. Crystallogr.* **2014**, *70* (6), 1549–1560. https://doi.org/10.1107/S1399004714005239.

(5) Chen, S.-C.; Huang, C.-H.; Lai, S.-J.; Liu, J.-S.; Fu, P.-K.; Tseng, S.-T.; Yang, C. S.; Lai, M.-C.; Ko, T.-P.; Chen, Y. Structure and Mechanism of an Antibiotics-Synthesizing 3-Hydroxykynurenine C-Methyltransferase. *Sci. Rep.* **2015**, *5* (1), 10100. https://doi.org/10.1038/srep10100.

(6) Robin, A. Y.; Giustini, C.; Graindorge, M.; Matringe, M.; Dumas, R. Crystal Structure of Norcoclaurine-6-O-Methyltransferase, a Key Rate-Limiting Step in the Synthesis of Benzylisoquinoline Alkaloids. *Plant J.* **2016**, *87* (6), 641–653. https://doi.org/10.1111/tpj.13225.

(7) Cabry, M. P.; Offen, W. A.; Saleh, P.; Li, Y.; Winzer, T.; Graham, I. A.; Davies, G. J. Structure of *Papaver Somniferum* O-Methyltransferase 1 Reveals Initiation of Noscapine Biosynthesis with Implications for Plant Natural Product Methylation. *ACS Catal.* **2019**, *9* (5), 3840–3848. https://doi.org/10.1021/acscatal.9b01038.

(8) Qiu, S.; Xu, D.; Xu, M.; Zhou, H.; Sun, N.; Zhang, L.; Zhao, M.; He, J.; Ran, T.; Sun, B.; Wang, W. Crystal Structures of PigF, an O-Methyltransferase Involved in the Prodigiosin Synthetic Pathway, Reveal an Induced-Fit Substrate-Recognition Mechanism. *IUCrJ* **2022**, *9* (2), 316–327. https://doi.org/10.1107/S2052252521011696.

(9) Li, C.-Y.; Crack, J. C.; Newton-Payne, S.; Murphy, A. R. J.; Chen, X.-L.; Pinchbeck, B. J.; Zhou, S.; Williams, B. T.; Peng, M.; Zhang, X.-H.; Chen, Y.; Le Brun, N. E.; Todd, J. D.; Zhang, Y.-Z. Mechanistic Insights into the Key Marine Dimethylsulfoniopropionate Synthesis Enzyme DsyB/DSYB. *mLife* **2022**, *1* (2), 114–130. https://doi.org/10.1002/mlf2.12030.

(10) Papadopoulos, J. S.; Agarwala, R. COBALT: Constraint-Based Alignment Tool for Multiple Protein Sequences. *Bioinformatics* **2007**, *23* (9), 1073–1079. https://doi.org/10.1093/bioinformatics/btm076.
